# Search for squarks and gluinos in events with hadronically decaying tau leptons, jets and missing transverse momentum in proton–proton collisions at $\sqrt{s}=13$ TeV recorded with the ATLAS detector

**DOI:** 10.1140/epjc/s10052-016-4481-2

**Published:** 2016-12-10

**Authors:** M. Aaboud, G. Aad, B. Abbott, J. Abdallah, O. Abdinov, B. Abeloos, R. Aben, O. S. AbouZeid, N. L. Abraham, H. Abramowicz, H. Abreu, R. Abreu, Y. Abulaiti, B. S. Acharya, L. Adamczyk, D. L. Adams, J. Adelman, S. Adomeit, T. Adye, A. A. Affolder, T. Agatonovic-Jovin, J. Agricola, J. A. Aguilar-Saavedra, S. P. Ahlen, F. Ahmadov, G. Aielli, H. Akerstedt, T. P. A. Åkesson, A. V. Akimov, G. L. Alberghi, J. Albert, S. Albrand, M. J. Alconada Verzini, M. Aleksa, I. N. Aleksandrov, C. Alexa, G. Alexander, T. Alexopoulos, M. Alhroob, B. Ali, M. Aliev, G. Alimonti, J. Alison, S. P. Alkire, B. M. M. Allbrooke, B. W. Allen, P. P. Allport, A. Aloisio, A. Alonso, F. Alonso, C. Alpigiani, A. A. Alshehri, M. Alstaty, B. Alvarez Gonzalez, D. Álvarez Piqueras, M. G. Alviggi, B. T. Amadio, K. Amako, Y. Amaral Coutinho, C. Amelung, D. Amidei, S. P. Amor Dos Santos, A. Amorim, S. Amoroso, G. Amundsen, C. Anastopoulos, L. S. Ancu, N. Andari, T. Andeen, C. F. Anders, G. Anders, J. K. Anders, K. J. Anderson, A. Andreazza, V. Andrei, S. Angelidakis, I. Angelozzi, P. Anger, A. Angerami, F. Anghinolfi, A. V. Anisenkov, N. Anjos, A. Annovi, C. Antel, M. Antonelli, A. Antonov, F. Anulli, M. Aoki, L. Aperio Bella, G. Arabidze, Y. Arai, J. P. Araque, A. T. H. Arce, F. A. Arduh, J-F. Arguin, S. Argyropoulos, M. Arik, A. J. Armbruster, L. J. Armitage, O. Arnaez, H. Arnold, M. Arratia, O. Arslan, A. Artamonov, G. Artoni, S. Artz, S. Asai, N. Asbah, A. Ashkenazi, B. Åsman, L. Asquith, K. Assamagan, R. Astalos, M. Atkinson, N. B. Atlay, K. Augsten, G. Avolio, B. Axen, M. K. Ayoub, G. Azuelos, M. A. Baak, A. E. Baas, M. J. Baca, H. Bachacou, K. Bachas, M. Backes, M. Backhaus, P. Bagiacchi, P. Bagnaia, Y. Bai, J. T. Baines, O. K. Baker, E. M. Baldin, P. Balek, T. Balestri, F. Balli, W. K. Balunas, E. Banas, Sw. Banerjee, A. A. E. Bannoura, L. Barak, E. L. Barberio, D. Barberis, M. Barbero, T. Barillari, M-S Barisits, T. Barklow, N. Barlow, S. L. Barnes, B. M. Barnett, R. M. Barnett, Z. Barnovska-Blenessy, A. Baroncelli, G. Barone, A. J. Barr, L. Barranco Navarro, F. Barreiro, J. Barreiro Guimarães da Costa, R. Bartoldus, A. E. Barton, P. Bartos, A. Basalaev, A. Bassalat, R. L. Bates, S. J. Batista, J. R. Batley, M. Battaglia, M. Bauce, F. Bauer, H. S. Bawa, J. B. Beacham, M. D. Beattie, T. Beau, P. H. Beauchemin, P. Bechtle, H. P. Beck, K. Becker, M. Becker, M. Beckingham, C. Becot, A. J. Beddall, A. Beddall, V. A. Bednyakov, M. Bedognetti, C. P. Bee, L. J. Beemster, T. A. Beermann, M. Begel, J. K. Behr, C. Belanger-Champagne, A. S. Bell, G. Bella, L. Bellagamba, A. Bellerive, M. Bellomo, K. Belotskiy, O. Beltramello, N. L. Belyaev, O. Benary, D. Benchekroun, M. Bender, K. Bendtz, N. Benekos, Y. Benhammou, E. Benhar Noccioli, J. Benitez, D. P. Benjamin, J. R. Bensinger, S. Bentvelsen, L. Beresford, M. Beretta, D. Berge, E. Bergeaas Kuutmann, N. Berger, J. Beringer, S. Berlendis, N. R. Bernard, C. Bernius, F. U. Bernlochner, T. Berry, P. Berta, C. Bertella, G. Bertoli, F. Bertolucci, I. A. Bertram, C. Bertsche, D. Bertsche, G. J. Besjes, O. Bessidskaia Bylund, M. Bessner, N. Besson, C. Betancourt, A. Bethani, S. Bethke, A. J. Bevan, R. M. Bianchi, L. Bianchini, M. Bianco, O. Biebel, D. Biedermann, R. Bielski, N. V. Biesuz, M. Biglietti, J. Bilbao De Mendizabal, T. R. V. Billoud, H. Bilokon, M. Bindi, S. Binet, A. Bingul, C. Bini, S. Biondi, T. Bisanz, D. M. Bjergaard, C. W. Black, J. E. Black, K. M. Black, D. Blackburn, R. E. Blair, J. -B. Blanchard, T. Blazek, I. Bloch, C. Blocker, A. Blue, W. Blum, U. Blumenschein, S. Blunier, G. J. Bobbink, V. S. Bobrovnikov, S. S. Bocchetta, A. Bocci, C. Bock, M. Boehler, D. Boerner, J. A. Bogaerts, D. Bogavac, A. G. Bogdanchikov, C. Bohm, V. Boisvert, P. Bokan, T. Bold, A. S. Boldyrev, M. Bomben, M. Bona, M. Boonekamp, A. Borisov, G. Borissov, J. Bortfeldt, D. Bortoletto, V. Bortolotto, K. Bos, D. Boscherini, M. Bosman, J. D. Bossio Sola, J. Boudreau, J. Bouffard, E. V. Bouhova-Thacker, D. Boumediene, C. Bourdarios, S. K. Boutle, A. Boveia, J. Boyd, I. R. Boyko, J. Bracinik, A. Brandt, G. Brandt, O. Brandt, U. Bratzler, B. Brau, J. E. Brau, H. M. Braun, W. D. Breaden Madden, K. Brendlinger, A. J. Brennan, L. Brenner, R. Brenner, S. Bressler, T. M. Bristow, D. Britton, D. Britzger, F. M. Brochu, I. Brock, R. Brock, G. Brooijmans, T. Brooks, W. K. Brooks, J. Brosamer, E. Brost, J. H Broughton, P. A. Bruckman de Renstrom, D. Bruncko, R. Bruneliere, A. Bruni, G. Bruni, L. S. Bruni, BH Brunt, M. Bruschi, N. Bruscino, P. Bryant, L. Bryngemark, T. Buanes, Q. Buat, P. Buchholz, A. G. Buckley, I. A. Budagov, F. Buehrer, M. K. Bugge, O. Bulekov, D. Bullock, H. Burckhart, S. Burdin, C. D. Burgard, B. Burghgrave, K. Burka, S. Burke, I. Burmeister, J. T. P. Burr, E. Busato, D. Büscher, V. Büscher, P. Bussey, J. M. Butler, C. M. Buttar, J. M. Butterworth, P. Butti, W. Buttinger, A. Buzatu, A. R. Buzykaev, S. Cabrera Urbán, D. Caforio, V. M. Cairo, O. Cakir, N. Calace, P. Calafiura, A. Calandri, G. Calderini, P. Calfayan, G. Callea, L. P. Caloba, S. Calvente Lopez, D. Calvet, S. Calvet, T. P. Calvet, R. Camacho Toro, S. Camarda, P. Camarri, D. Cameron, R. Caminal Armadans, C. Camincher, S. Campana, M. Campanelli, A. Camplani, A. Campoverde, V. Canale, A. Canepa, M. Cano Bret, J. Cantero, T. Cao, M. D. M. Capeans Garrido, I. Caprini, M. Caprini, M. Capua, R. M. Carbone, R. Cardarelli, F. Cardillo, I. Carli, T. Carli, G. Carlino, L. Carminati, S. Caron, E. Carquin, G. D. Carrillo-Montoya, J. R. Carter, J. Carvalho, D. Casadei, M. P. Casado, M. Casolino, D. W. Casper, E. Castaneda-Miranda, R. Castelijn, A. Castelli, V. Castillo Gimenez, N. F. Castro, A. Catinaccio, J. R. Catmore, A. Cattai, J. Caudron, V. Cavaliere, E. Cavallaro, D. Cavalli, M. Cavalli-Sforza, V. Cavasinni, F. Ceradini, L. Cerda Alberich, B. C. Cerio, A. S. Cerqueira, A. Cerri, L. Cerrito, F. Cerutti, M. Cerv, A. Cervelli, S. A. Cetin, A. Chafaq, D. Chakraborty, S. K. Chan, Y. L. Chan, P. Chang, J. D. Chapman, D. G. Charlton, A. Chatterjee, C. C. Chau, C. A. Chavez Barajas, S. Che, S. Cheatham, A. Chegwidden, S. Chekanov, S. V. Chekulaev, G. A. Chelkov, M. A. Chelstowska, C. Chen, H. Chen, K. Chen, S. Chen, S. Chen, X. Chen, Y. Chen, H. C. Cheng, H. J Cheng, Y. Cheng, A. Cheplakov, E. Cheremushkina, R. Cherkaoui El Moursli, V. Chernyatin, E. Cheu, L. Chevalier, V. Chiarella, G. Chiarelli, G. Chiodini, A. S. Chisholm, A. Chitan, M. V. Chizhov, K. Choi, A. R. Chomont, S. Chouridou, B. K. B. Chow, V. Christodoulou, D. Chromek-Burckhart, J. Chudoba, A. J. Chuinard, J. J. Chwastowski, L. Chytka, G. Ciapetti, A. K. Ciftci, D. Cinca, V. Cindro, I. A. Cioara, C. Ciocca, A. Ciocio, F. Cirotto, Z. H. Citron, M. Citterio, M. Ciubancan, A. Clark, B. L. Clark, M. R. Clark, P. J. Clark, R. N. Clarke, C. Clement, Y. Coadou, M. Cobal, A. Coccaro, J. Cochran, L. Colasurdo, B. Cole, A. P. Colijn, J. Collot, T. Colombo, G. Compostella, P. Conde Muiño, E. Coniavitis, S. H. Connell, I. A. Connelly, V. Consorti, S. Constantinescu, G. Conti, F. Conventi, M. Cooke, B. D. Cooper, A. M. Cooper-Sarkar, K. J. R. Cormier, T. Cornelissen, M. Corradi, F. Corriveau, A. Corso-Radu, A. Cortes-Gonzalez, G. Cortiana, G. Costa, M. J. Costa, D. Costanzo, G. Cottin, G. Cowan, B. E. Cox, K. Cranmer, S. J. Crawley, G. Cree, S. Crépé-Renaudin, F. Crescioli, W. A. Cribbs, M. Crispin Ortuzar, M. Cristinziani, V. Croft, G. Crosetti, A. Cueto, T. Cuhadar Donszelmann, J. Cummings, M. Curatolo, J. Cúth, H. Czirr, P. Czodrowski, G. D’amen, S. D’Auria, M. D’Onofrio, M. J. Da Cunha Sargedas De Sousa, C. Da Via, W. Dabrowski, T. Dado, T. Dai, O. Dale, F. Dallaire, C. Dallapiccola, M. Dam, J. R. Dandoy, N. P. Dang, A. C. Daniells, N. S. Dann, M. Danninger, M. Dano Hoffmann, V. Dao, G. Darbo, S. Darmora, J. Dassoulas, A. Dattagupta, W. Davey, C. David, T. Davidek, M. Davies, P. Davison, E. Dawe, I. Dawson, K. De, R. de Asmundis, A. De Benedetti, S. De Castro, S. De Cecco, N. De Groot, P. de Jong, H. De la Torre, F. De Lorenzi, A. De Maria, D. De Pedis, A. De Salvo, U. De Sanctis, A. De Santo, J. B. De Vivie De Regie, W. J. Dearnaley, R. Debbe, C. Debenedetti, D. V. Dedovich, N. Dehghanian, I. Deigaard, M. Del Gaudio, J. Del Peso, T. Del Prete, D. Delgove, F. Deliot, C. M. Delitzsch, A. Dell’Acqua, L. Dell’Asta, M. Dell’Orso, M. Della Pietra, D. della Volpe, M. Delmastro, P. A. Delsart, D. A. DeMarco, S. Demers, M. Demichev, A. Demilly, S. P. Denisov, D. Denysiuk, D. Derendarz, J. E. Derkaoui, F. Derue, P. Dervan, K. Desch, C. Deterre, K. Dette, P. O. Deviveiros, A. Dewhurst, S. Dhaliwal, A. Di Ciaccio, L. Di Ciaccio, W. K. Di Clemente, C. Di Donato, A. Di Girolamo, B. Di Girolamo, B. Di Micco, R. Di Nardo, A. Di Simone, R. Di Sipio, D. Di Valentino, C. Diaconu, M. Diamond, F. A. Dias, M. A. Diaz, E. B. Diehl, J. Dietrich, S. Díez Cornell, A. Dimitrievska, J. Dingfelder, P. Dita, S. Dita, F. Dittus, F. Djama, T. Djobava, J. I. Djuvsland, M. A. B. do Vale, D. Dobos, M. Dobre, C. Doglioni, J. Dolejsi, Z. Dolezal, M. Donadelli, S. Donati, P. Dondero, J. Donini, J. Dopke, A. Doria, M. T. Dova, A. T. Doyle, E. Drechsler, M. Dris, Y. Du, J. Duarte-Campderros, E. Duchovni, G. Duckeck, O. A. Ducu, D. Duda, A. Dudarev, A. Chr. Dudder, E. M. Duffield, L. Duflot, M. Dührssen, M. Dumancic, M. Dunford, H. Duran Yildiz, M. Düren, A. Durglishvili, D. Duschinger, B. Dutta, M. Dyndal, C. Eckardt, K. M. Ecker, R. C. Edgar, N. C. Edwards, T. Eifert, G. Eigen, K. Einsweiler, T. Ekelof, M. El Kacimi, V. Ellajosyula, M. Ellert, S. Elles, F. Ellinghaus, A. A. Elliot, N. Ellis, J. Elmsheuser, M. Elsing, D. Emeliyanov, Y. Enari, O. C. Endner, J. S. Ennis, J. Erdmann, A. Ereditato, G. Ernis, J. Ernst, M. Ernst, S. Errede, E. Ertel, M. Escalier, H. Esch, C. Escobar, B. Esposito, A. I. Etienvre, E. Etzion, H. Evans, A. Ezhilov, F. Fabbri, L. Fabbri, G. Facini, R. M. Fakhrutdinov, S. Falciano, R. J. Falla, J. Faltova, Y. Fang, M. Fanti, A. Farbin, A. Farilla, C. Farina, E. M. Farina, T. Farooque, S. Farrell, S. M. Farrington, P. Farthouat, F. Fassi, P. Fassnacht, D. Fassouliotis, M. Faucci Giannelli, A. Favareto, W. J. Fawcett, L. Fayard, O. L. Fedin, W. Fedorko, S. Feigl, L. Feligioni, C. Feng, E. J. Feng, H. Feng, A. B. Fenyuk, L. Feremenga, P. Fernandez Martinez, S. Fernandez Perez, J. Ferrando, A. Ferrari, P. Ferrari, R. Ferrari, D. E. Ferreira de Lima, A. Ferrer, D. Ferrere, C. Ferretti, A. Ferretto Parodi, F. Fiedler, A. Filipčič, M. Filipuzzi, F. Filthaut, M. Fincke-Keeler, K. D. Finelli, M. C. N. Fiolhais, L. Fiorini, A. Firan, A. Fischer, C. Fischer, J. Fischer, W. C. Fisher, N. Flaschel, I. Fleck, P. Fleischmann, G. T. Fletcher, R. R. M. Fletcher, T. Flick, L. R. Flores Castillo, M. J. Flowerdew, G. T. Forcolin, A. Formica, A. Forti, A. G. Foster, D. Fournier, H. Fox, S. Fracchia, P. Francavilla, M. Franchini, D. Francis, L. Franconi, M. Franklin, M. Frate, M. Fraternali, D. Freeborn, S. M. Fressard-Batraneanu, F. Friedrich, D. Froidevaux, J. A. Frost, C. Fukunaga, E. Fullana Torregrosa, T. Fusayasu, J. Fuster, C. Gabaldon, O. Gabizon, A. Gabrielli, A. Gabrielli, G. P. Gach, S. Gadatsch, S. Gadomski, G. Gagliardi, L. G. Gagnon, P. Gagnon, C. Galea, B. Galhardo, E. J. Gallas, B. J. Gallop, P. Gallus, G. Galster, K. K. Gan, J. Gao, Y. Gao, Y. S. Gao, F. M. Garay Walls, C. García, J. E. García Navarro, M. Garcia-Sciveres, R. W. Gardner, N. Garelli, V. Garonne, A. Gascon Bravo, K. Gasnikova, C. Gatti, A. Gaudiello, G. Gaudio, L. Gauthier, I. L. Gavrilenko, C. Gay, G. Gaycken, E. N. Gazis, Z. Gecse, C. N. P. Gee, Ch. Geich-Gimbel, M. Geisen, M. P. Geisler, K. Gellerstedt, C. Gemme, M. H. Genest, C. Geng, S. Gentile, C. Gentsos, S. George, D. Gerbaudo, A. Gershon, S. Ghasemi, M. Ghneimat, B. Giacobbe, S. Giagu, P. Giannetti, B. Gibbard, S. M. Gibson, M. Gignac, M. Gilchriese, T. P. S. Gillam, D. Gillberg, G. Gilles, D. M. Gingrich, N. Giokaris, M. P. Giordani, F. M. Giorgi, F. M. Giorgi, P. F. Giraud, P. Giromini, D. Giugni, F. Giuli, C. Giuliani, M. Giulini, B. K. Gjelsten, S. Gkaitatzis, I. Gkialas, E. L. Gkougkousis, L. K. Gladilin, C. Glasman, J. Glatzer, P. C. F. Glaysher, A. Glazov, M. Goblirsch-Kolb, J. Godlewski, S. Goldfarb, T. Golling, D. Golubkov, A. Gomes, R. Gonçalo, J. Goncalves Pinto Firmino Da Costa, G. Gonella, L. Gonella, A. Gongadze, S. González de la Hoz, G. Gonzalez Parra, S. Gonzalez-Sevilla, L. Goossens, P. A. Gorbounov, H. A. Gordon, I. Gorelov, B. Gorini, E. Gorini, A. Gorišek, E. Gornicki, A. T. Goshaw, C. Gössling, M. I. Gostkin, C. R. Goudet, D. Goujdami, A. G. Goussiou, N. Govender, E. Gozani, L. Graber, I. Grabowska-Bold, P. O. J. Gradin, P. Grafström, J. Gramling, E. Gramstad, S. Grancagnolo, V. Gratchev, P. M. Gravila, H. M. Gray, E. Graziani, Z. D. Greenwood, C. Grefe, K. Gregersen, I. M. Gregor, P. Grenier, K. Grevtsov, J. Griffiths, A. A. Grillo, K. Grimm, S. Grinstein, Ph. Gris, J. -F. Grivaz, S. Groh, J. P. Grohs, E. Gross, J. Grosse-Knetter, G. C. Grossi, Z. J. Grout, L. Guan, W. Guan, J. Guenther, F. Guescini, D. Guest, O. Gueta, E. Guido, T. Guillemin, S. Guindon, U. Gul, C. Gumpert, J. Guo, Y. Guo, R. Gupta, S. Gupta, G. Gustavino, P. Gutierrez, N. G. Gutierrez Ortiz, C. Gutschow, C. Guyot, C. Gwenlan, C. B. Gwilliam, A. Haas, C. Haber, H. K. Hadavand, N. Haddad, A. Hadef, S. Hageböck, Z. Hajduk, H. Hakobyan, M. Haleem, J. Haley, G. Halladjian, G. D. Hallewell, K. Hamacher, P. Hamal, K. Hamano, A. Hamilton, G. N. Hamity, P. G. Hamnett, L. Han, K. Hanagaki, K. Hanawa, M. Hance, B. Haney, P. Hanke, R. Hanna, J. B. Hansen, J. D. Hansen, M. C. Hansen, P. H. Hansen, K. Hara, A. S. Hard, T. Harenberg, F. Hariri, S. Harkusha, R. D. Harrington, P. F. Harrison, F. Hartjes, N. M. Hartmann, M. Hasegawa, Y. Hasegawa, A. Hasib, S. Hassani, S. Haug, R. Hauser, L. Hauswald, M. Havranek, C. M. Hawkes, R. J. Hawkings, D. Hayakawa, D. Hayden, C. P. Hays, J. M. Hays, H. S. Hayward, S. J. Haywood, S. J. Head, T. Heck, V. Hedberg, L. Heelan, S. Heim, T. Heim, B. Heinemann, J. J. Heinrich, L. Heinrich, C. Heinz, J. Hejbal, L. Helary, S. Hellman, C. Helsens, J. Henderson, R. C. W. Henderson, Y. Heng, S. Henkelmann, A. M. Henriques Correia, S. Henrot-Versille, G. H. Herbert, H. Herde, V. Herget, Y. Hernández Jiménez, G. Herten, R. Hertenberger, L. Hervas, G. G. Hesketh, N. P. Hessey, J. W. Hetherly, R. Hickling, E. Higón-Rodriguez, E. Hill, J. C. Hill, K. H. Hiller, S. J. Hillier, I. Hinchliffe, E. Hines, R. R. Hinman, M. Hirose, D. Hirschbuehl, J. Hobbs, N. Hod, M. C. Hodgkinson, P. Hodgson, A. Hoecker, M. R. Hoeferkamp, F. Hoenig, D. Hohn, T. R. Holmes, M. Homann, T. Honda, T. M. Hong, B. H. Hooberman, W. H. Hopkins, Y. Horii, A. J. Horton, J-Y. Hostachy, S. Hou, A. Hoummada, J. Howarth, J. Hoya, M. Hrabovsky, I. Hristova, J. Hrivnac, T. Hryn’ova, A. Hrynevich, C. Hsu, P. J. Hsu, S. -C. Hsu, D. Hu, Q. Hu, S. Hu, Y. Huang, Z. Hubacek, F. Hubaut, F. Huegging, T. B. Huffman, E. W. Hughes, G. Hughes, M. Huhtinen, P. Huo, N. Huseynov, J. Huston, J. Huth, G. Iacobucci, G. Iakovidis, I. Ibragimov, L. Iconomidou-Fayard, E. Ideal, Z. Idrissi, P. Iengo, O. Igonkina, T. Iizawa, Y. Ikegami, M. Ikeno, Y. Ilchenko, D. Iliadis, N. Ilic, T. Ince, G. Introzzi, P. Ioannou, M. Iodice, K. Iordanidou, V. Ippolito, N. Ishijima, M. Ishino, M. Ishitsuka, R. Ishmukhametov, C. Issever, S. Istin, F. Ito, J. M. Iturbe Ponce, R. Iuppa, W. Iwanski, H. Iwasaki, J. M. Izen, V. Izzo, S. Jabbar, B. Jackson, P. Jackson, V. Jain, K. B. Jakobi, K. Jakobs, S. Jakobsen, T. Jakoubek, D. O. Jamin, D. K. Jana, R. Jansky, J. Janssen, M. Janus, G. Jarlskog, N. Javadov, T. Javůrek, F. Jeanneau, L. Jeanty, G. -Y. Jeng, D. Jennens, P. Jenni, C. Jeske, S. Jézéquel, H. Ji, J. Jia, H. Jiang, Y. Jiang, S. Jiggins, J. Jimenez Pena, S. Jin, A. Jinaru, O. Jinnouchi, H. Jivan, P. Johansson, K. A. Johns, W. J. Johnson, K. Jon-And, G. Jones, R. W. L. Jones, S. Jones, T. J. Jones, J. Jongmanns, P. M. Jorge, J. Jovicevic, X. Ju, A. Juste Rozas, M. K. Köhler, A. Kaczmarska, M. Kado, H. Kagan, M. Kagan, S. J. Kahn, T. Kaji, E. Kajomovitz, C. W. Kalderon, A. Kaluza, S. Kama, A. Kamenshchikov, N. Kanaya, S. Kaneti, L. Kanjir, V. A. Kantserov, J. Kanzaki, B. Kaplan, L. S. Kaplan, A. Kapliy, D. Kar, K. Karakostas, A. Karamaoun, N. Karastathis, M. J. Kareem, E. Karentzos, M. Karnevskiy, S. N. Karpov, Z. M. Karpova, K. Karthik, V. Kartvelishvili, A. N. Karyukhin, K. Kasahara, L. Kashif, R. D. Kass, A. Kastanas, Y. Kataoka, C. Kato, A. Katre, J. Katzy, K. Kawade, K. Kawagoe, T. Kawamoto, G. Kawamura, V. F. Kazanin, R. Keeler, R. Kehoe, J. S. Keller, J. J. Kempster, H. Keoshkerian, O. Kepka, B. P. Kerševan, S. Kersten, R. A. Keyes, M. Khader, F. Khalil-zada, A. Khanov, A. G. Kharlamov, T. Kharlamova, T. J. Khoo, V. Khovanskiy, E. Khramov, J. Khubua, S. Kido, C. R. Kilby, H. Y. Kim, S. H. Kim, Y. K. Kim, N. Kimura, O. M. Kind, B. T. King, M. King, J. Kirk, A. E. Kiryunin, T. Kishimoto, D. Kisielewska, F. Kiss, K. Kiuchi, O. Kivernyk, E. Kladiva, M. H. Klein, M. Klein, U. Klein, K. Kleinknecht, P. Klimek, A. Klimentov, R. Klingenberg, J. A. Klinger, T. Klioutchnikova, E. -E. Kluge, P. Kluit, S. Kluth, J. Knapik, E. Kneringer, E. B. F. G. Knoops, A. Knue, A. Kobayashi, D. Kobayashi, T. Kobayashi, M. Kobel, M. Kocian, P. Kodys, N. M. Koehler, T. Koffas, E. Koffeman, T. Koi, H. Kolanoski, M. Kolb, I. Koletsou, A. A. Komar, Y. Komori, T. Kondo, N. Kondrashova, K. Köneke, A. C. König, T. Kono, R. Konoplich, N. Konstantinidis, R. Kopeliansky, S. Koperny, L. Köpke, A. K. Kopp, K. Korcyl, K. Kordas, A. Korn, A. A. Korol, I. Korolkov, E. V. Korolkova, O. Kortner, S. Kortner, T. Kosek, V. V. Kostyukhin, A. Kotwal, A. Kourkoumeli-Charalampidi, C. Kourkoumelis, V. Kouskoura, A. B. Kowalewska, R. Kowalewski, T. Z. Kowalski, C. Kozakai, W. Kozanecki, A. S. Kozhin, V. A. Kramarenko, G. Kramberger, D. Krasnopevtsev, M. W. Krasny, A. Krasznahorkay, A. Kravchenko, M. Kretz, J. Kretzschmar, K. Kreutzfeldt, P. Krieger, K. Krizka, K. Kroeninger, H. Kroha, J. Kroll, J. Kroseberg, J. Krstic, U. Kruchonak, H. Krüger, N. Krumnack, A. Kruse, M. C. Kruse, M. Kruskal, T. Kubota, H. Kucuk, S. Kuday, J. T. Kuechler, S. Kuehn, A. Kugel, F. Kuger, A. Kuhl, T. Kuhl, V. Kukhtin, R. Kukla, Y. Kulchitsky, S. Kuleshov, M. Kuna, T. Kunigo, A. Kupco, H. Kurashige, Y. A. Kurochkin, V. Kus, E. S. Kuwertz, M. Kuze, J. Kvita, T. Kwan, D. Kyriazopoulos, A. La Rosa, J. L. La Rosa Navarro, L. La Rotonda, C. Lacasta, F. Lacava, J. Lacey, H. Lacker, D. Lacour, V. R. Lacuesta, E. Ladygin, R. Lafaye, B. Laforge, T. Lagouri, S. Lai, S. Lammers, W. Lampl, E. Lançon, U. Landgraf, M. P. J. Landon, M. C. Lanfermann, V. S. Lang, J. C. Lange, A. J. Lankford, F. Lanni, K. Lantzsch, A. Lanza, S. Laplace, C. Lapoire, J. F. Laporte, T. Lari, F. Lasagni Manghi, M. Lassnig, P. Laurelli, W. Lavrijsen, A. T. Law, P. Laycock, T. Lazovich, M. Lazzaroni, B. Le, O. Le Dortz, E. Le Guirriec, E. P. Le Quilleuc, M. LeBlanc, T. LeCompte, F. Ledroit-Guillon, C. A. Lee, S. C. Lee, L. Lee, B. Lefebvre, G. Lefebvre, M. Lefebvre, F. Legger, C. Leggett, A. Lehan, G. Lehmann Miotto, X. Lei, W. A. Leight, A. Leisos, A. G. Leister, M. A. L. Leite, R. Leitner, D. Lellouch, B. Lemmer, K. J. C. Leney, T. Lenz, B. Lenzi, R. Leone, S. Leone, C. Leonidopoulos, S. Leontsinis, G. Lerner, C. Leroy, A. A. J. Lesage, C. G. Lester, M. Levchenko, J. Levêque, D. Levin, L. J. Levinson, M. Levy, D. Lewis, A. M. Leyko, M. Leyton, B. Li, C. Li, H. Li, H. L. Li, L. Li, L. Li, Q. Li, S. Li, X. Li, Y. Li, Z. Liang, B. Liberti, A. Liblong, P. Lichard, K. Lie, J. Liebal, W. Liebig, A. Limosani, S. C. Lin, T. H. Lin, B. E. Lindquist, A. E. Lionti, E. Lipeles, A. Lipniacka, M. Lisovyi, T. M. Liss, A. Lister, A. M. Litke, B. Liu, D. Liu, H. Liu, H. Liu, J. Liu, J. B. Liu, K. Liu, L. Liu, M. Liu, M. Liu, Y. L. Liu, Y. Liu, M. Livan, A. Lleres, J. Llorente Merino, S. L. Lloyd, F. Lo Sterzo, E. M. Lobodzinska, P. Loch, W. S. Lockman, F. K. Loebinger, A. E. Loevschall-Jensen, K. M. Loew, A. Loginov, T. Lohse, K. Lohwasser, M. Lokajicek, B. A. Long, J. D. Long, R. E. Long, L. Longo, K. A. Looper, D. Lopez Mateos, B. Lopez Paredes, I. Lopez Paz, A. Lopez Solis, J. Lorenz, N. Lorenzo Martinez, M. Losada, P. J. Lösel, X. Lou, A. Lounis, J. Love, P. A. Love, H. Lu, N. Lu, H. J. Lubatti, C. Luci, A. Lucotte, C. Luedtke, F. Luehring, W. Lukas, L. Luminari, O. Lundberg, B. Lund-Jensen, P. M. Luzi, D. Lynn, R. Lysak, E. Lytken, V. Lyubushkin, H. Ma, L. L. Ma, Y. Ma, G. Maccarrone, A. Macchiolo, C. M. Macdonald, B. Maček, J. Machado Miguens, D. Madaffari, R. Madar, H. J. Maddocks, W. F. Mader, A. Madsen, J. Maeda, S. Maeland, T. Maeno, A. Maevskiy, E. Magradze, J. Mahlstedt, C. Maiani, C. Maidantchik, A. A. Maier, T. Maier, A. Maio, S. Majewski, Y. Makida, N. Makovec, B. Malaescu, Pa. Malecki, V. P. Maleev, F. Malek, U. Mallik, D. Malon, C. Malone, C. Malone, S. Maltezos, S. Malyukov, J. Mamuzic, G. Mancini, L. Mandelli, I. Mandić, J. Maneira, L. Manhaes de Andrade Filho, J. Manjarres Ramos, A. Mann, A. Manousos, B. Mansoulie, J. D. Mansour, R. Mantifel, M. Mantoani, S. Manzoni, L. Mapelli, G. Marceca, L. March, G. Marchiori, M. Marcisovsky, M. Marjanovic, D. E. Marley, F. Marroquim, S. P. Marsden, Z. Marshall, S. Marti-Garcia, B. Martin, T. A. Martin, V. J. Martin, B. Martin dit Latour, M. Martinez, V. I. Martinez Outschoorn, S. Martin-Haugh, V. S. Martoiu, A. C. Martyniuk, M. Marx, A. Marzin, L. Masetti, T. Mashimo, R. Mashinistov, J. Masik, A. L. Maslennikov, I. Massa, L. Massa, P. Mastrandrea, A. Mastroberardino, T. Masubuchi, P. Mättig, J. Mattmann, J. Maurer, S. J. Maxfield, D. A. Maximov, R. Mazini, S. M. Mazza, N. C. Mc Fadden, G. Mc Goldrick, S. P. Mc Kee, A. McCarn, R. L. McCarthy, T. G. McCarthy, L. I. McClymont, E. F. McDonald, J. A. Mcfayden, G. Mchedlidze, S. J. McMahon, R. A. McPherson, M. Medinnis, S. Meehan, S. Mehlhase, A. Mehta, K. Meier, C. Meineck, B. Meirose, D. Melini, B. R. Mellado Garcia, M. Melo, F. Meloni, A. Mengarelli, S. Menke, E. Meoni, S. Mergelmeyer, P. Mermod, L. Merola, C. Meroni, F. S. Merritt, A. Messina, J. Metcalfe, A. S. Mete, C. Meyer, C. Meyer, J-P. Meyer, J. Meyer, H. Meyer Zu Theenhausen, F. Miano, R. P. Middleton, S. Miglioranzi, L. Mijović, G. Mikenberg, M. Mikestikova, M. Mikuž, M. Milesi, A. Milic, D. W. Miller, C. Mills, A. Milov, D. A. Milstead, A. A. Minaenko, Y. Minami, I. A. Minashvili, A. I. Mincer, B. Mindur, M. Mineev, Y. Minegishi, Y. Ming, L. M. Mir, K. P. Mistry, T. Mitani, J. Mitrevski, V. A. Mitsou, A. Miucci, P. S. Miyagawa, J. U. Mjörnmark, M. Mlynarikova, T. Moa, K. Mochizuki, S. Mohapatra, S. Molander, R. Moles-Valls, R. Monden, M. C. Mondragon, K. Mönig, J. Monk, E. Monnier, A. Montalbano, J. Montejo Berlingen, F. Monticelli, S. Monzani, R. W. Moore, N. Morange, D. Moreno, M. Moreno Llácer, P. Morettini, S. Morgenstern, D. Mori, T. Mori, M. Morii, M. Morinaga, V. Morisbak, S. Moritz, A. K. Morley, G. Mornacchi, J. D. Morris, S. S. Mortensen, L. Morvaj, M. Mosidze, J. Moss, K. Motohashi, R. Mount, E. Mountricha, E. J. W. Moyse, S. Muanza, R. D. Mudd, F. Mueller, J. Mueller, R. S. P. Mueller, T. Mueller, D. Muenstermann, P. Mullen, G. A. Mullier, F. J. Munoz Sanchez, J. A. Murillo Quijada, W. J. Murray, H. Musheghyan, M. Muškinja, A. G. Myagkov, M. Myska, B. P. Nachman, O. Nackenhorst, K. Nagai, R. Nagai, K. Nagano, Y. Nagasaka, K. Nagata, M. Nagel, E. Nagy, A. M. Nairz, Y. Nakahama, K. Nakamura, T. Nakamura, I. Nakano, H. Namasivayam, R. F. Naranjo Garcia, R. Narayan, D. I. Narrias Villar, I. Naryshkin, T. Naumann, G. Navarro, R. Nayyar, H. A. Neal, P. Yu. Nechaeva, T. J. Neep, A. Negri, M. Negrini, S. Nektarijevic, C. Nellist, A. Nelson, S. Nemecek, P. Nemethy, A. A. Nepomuceno, M. Nessi, M. S. Neubauer, M. Neumann, R. M. Neves, P. Nevski, P. R. Newman, D. H. Nguyen, T. Nguyen Manh, R. B. Nickerson, R. Nicolaidou, J. Nielsen, A. Nikiforov, V. Nikolaenko, I. Nikolic-Audit, K. Nikolopoulos, J. K. Nilsen, P. Nilsson, Y. Ninomiya, A. Nisati, R. Nisius, T. Nobe, M. Nomachi, I. Nomidis, T. Nooney, S. Norberg, M. Nordberg, N. Norjoharuddeen, O. Novgorodova, S. Nowak, M. Nozaki, L. Nozka, K. Ntekas, E. Nurse, F. Nuti, F. O’grady, D. C. O’Neil, A. A. O’Rourke, V. O’Shea, F. G. Oakham, H. Oberlack, T. Obermann, J. Ocariz, A. Ochi, I. Ochoa, J. P. Ochoa-Ricoux, S. Oda, S. Odaka, H. Ogren, A. Oh, S. H. Oh, C. C. Ohm, H. Ohman, H. Oide, H. Okawa, Y. Okumura, T. Okuyama, A. Olariu, L. F. Oleiro Seabra, S. A. Olivares Pino, D. Oliveira Damazio, A. Olszewski, J. Olszowska, A. Onofre, K. Onogi, P. U. E. Onyisi, M. J. Oreglia, Y. Oren, D. Orestano, N. Orlando, R. S. Orr, B. Osculati, R. Ospanov, G. Otero y Garzon, H. Otono, M. Ouchrif, F. Ould-Saada, A. Ouraou, K. P. Oussoren, Q. Ouyang, M. Owen, R. E. Owen, V. E. Ozcan, N. Ozturk, K. Pachal, A. Pacheco Pages, L. Pacheco Rodriguez, C. Padilla Aranda, M. Pagáčová, S. Pagan Griso, M. Paganini, F. Paige, P. Pais, K. Pajchel, G. Palacino, S. Palazzo, S. Palestini, M. Palka, D. Pallin, E. St. Panagiotopoulou, C. E. Pandini, J. G. Panduro Vazquez, P. Pani, S. Panitkin, D. Pantea, L. Paolozzi, Th. D. Papadopoulou, K. Papageorgiou, A. Paramonov, D. Paredes Hernandez, A. J. Parker, M. A. Parker, K. A. Parker, F. Parodi, J. A. Parsons, U. Parzefall, V. R. Pascuzzi, E. Pasqualucci, S. Passaggio, Fr. Pastore, G. Pásztor, S. Pataraia, J. R. Pater, T. Pauly, J. Pearce, B. Pearson, L. E. Pedersen, M. Pedersen, S. Pedraza Lopez, R. Pedro, S. V. Peleganchuk, O. Penc, C. Peng, H. Peng, J. Penwell, B. S. Peralva, M. M. Perego, D. V. Perepelitsa, E. Perez Codina, L. Perini, H. Pernegger, S. Perrella, R. Peschke, V. D. Peshekhonov, K. Peters, R. F. Y. Peters, B. A. Petersen, T. C. Petersen, E. Petit, A. Petridis, C. Petridou, P. Petroff, E. Petrolo, M. Petrov, F. Petrucci, N. E. Pettersson, A. Peyaud, R. Pezoa, P. W. Phillips, G. Piacquadio, E. Pianori, A. Picazio, E. Piccaro, M. Piccinini, M. A. Pickering, R. Piegaia, J. E. Pilcher, A. D. Pilkington, A. W. J. Pin, M. Pinamonti, J. L. Pinfold, A. Pingel, S. Pires, H. Pirumov, M. Pitt, L. Plazak, M. -A. Pleier, V. Pleskot, E. Plotnikova, P. Plucinski, D. Pluth, R. Poettgen, L. Poggioli, D. Pohl, G. Polesello, A. Poley, A. Policicchio, R. Polifka, A. Polini, C. S. Pollard, V. Polychronakos, K. Pommès, L. Pontecorvo, B. G. Pope, G. A. Popeneciu, A. Poppleton, S. Pospisil, K. Potamianos, I. N. Potrap, C. J. Potter, C. T. Potter, G. Poulard, J. Poveda, V. Pozdnyakov, M. E. Pozo Astigarraga, P. Pralavorio, A. Pranko, S. Prell, D. Price, L. E. Price, M. Primavera, S. Prince, K. Prokofiev, F. Prokoshin, S. Protopopescu, J. Proudfoot, M. Przybycien, D. Puddu, M. Purohit, P. Puzo, J. Qian, G. Qin, Y. Qin, A. Quadt, W. B. Quayle, M. Queitsch-Maitland, D. Quilty, S. Raddum, V. Radeka, V. Radescu, S. K. Radhakrishnan, P. Radloff, P. Rados, F. Ragusa, G. Rahal, J. A. Raine, S. Rajagopalan, M. Rammensee, C. Rangel-Smith, M. G. Ratti, F. Rauscher, S. Rave, T. Ravenscroft, I. Ravinovich, M. Raymond, A. L. Read, N. P. Readioff, M. Reale, D. M. Rebuzzi, A. Redelbach, G. Redlinger, R. Reece, K. Reeves, L. Rehnisch, J. Reichert, A. Reiss, C. Rembser, H. Ren, M. Rescigno, S. Resconi, O. L. Rezanova, P. Reznicek, R. Rezvani, R. Richter, S. Richter, E. Richter-Was, O. Ricken, M. Ridel, P. Rieck, C. J. Riegel, J. Rieger, O. Rifki, M. Rijssenbeek, A. Rimoldi, M. Rimoldi, L. Rinaldi, B. Ristić, E. Ritsch, I. Riu, F. Rizatdinova, E. Rizvi, C. Rizzi, S. H. Robertson, A. Robichaud-Veronneau, D. Robinson, J. E. M. Robinson, A. Robson, C. Roda, Y. Rodina, A. Rodriguez Perez, D. Rodriguez Rodriguez, S. Roe, C. S. Rogan, O. Røhne, A. Romaniouk, M. Romano, S. M. Romano Saez, E. Romero Adam, N. Rompotis, M. Ronzani, L. Roos, E. Ros, S. Rosati, K. Rosbach, P. Rose, N. -A. Rosien, V. Rossetti, E. Rossi, L. P. Rossi, J. H. N. Rosten, R. Rosten, M. Rotaru, I. Roth, J. Rothberg, D. Rousseau, A. Rozanov, Y. Rozen, X. Ruan, F. Rubbo, M. S. Rudolph, F. Rühr, A. Ruiz-Martinez, Z. Rurikova, N. A. Rusakovich, A. Ruschke, H. L. Russell, J. P. Rutherfoord, N. Ruthmann, Y. F. Ryabov, M. Rybar, G. Rybkin, S. Ryu, A. Ryzhov, G. F. Rzehorz, A. F. Saavedra, G. Sabato, S. Sacerdoti, H. F-W. Sadrozinski, R. Sadykov, F. Safai Tehrani, P. Saha, M. Sahinsoy, M. Saimpert, T. Saito, H. Sakamoto, Y. Sakurai, G. Salamanna, A. Salamon, J. E. Salazar Loyola, D. Salek, P. H. Sales De Bruin, D. Salihagic, A. Salnikov, J. Salt, D. Salvatore, F. Salvatore, A. Salvucci, A. Salzburger, D. Sammel, D. Sampsonidis, A. Sanchez, J. Sánchez, V. Sanchez Martinez, H. Sandaker, R. L. Sandbach, H. G. Sander, M. Sandhoff, C. Sandoval, D. P. C. Sankey, M. Sannino, A. Sansoni, C. Santoni, R. Santonico, H. Santos, I. Santoyo Castillo, K. Sapp, A. Sapronov, J. G. Saraiva, B. Sarrazin, O. Sasaki, K. Sato, E. Sauvan, G. Savage, P. Savard, N. Savic, C. Sawyer, L. Sawyer, J. Saxon, C. Sbarra, A. Sbrizzi, T. Scanlon, D. A. Scannicchio, M. Scarcella, V. Scarfone, J. Schaarschmidt, P. Schacht, B. M. Schachtner, D. Schaefer, L. Schaefer, R. Schaefer, J. Schaeffer, S. Schaepe, S. Schaetzel, U. Schäfer, A. C. Schaffer, D. Schaile, R. D. Schamberger, V. Scharf, V. A. Schegelsky, D. Scheirich, M. Schernau, C. Schiavi, S. Schier, C. Schillo, M. Schioppa, S. Schlenker, K. R. Schmidt-Sommerfeld, K. Schmieden, C. Schmitt, S. Schmitt, S. Schmitz, B. Schneider, U. Schnoor, L. Schoeffel, A. Schoening, B. D. Schoenrock, E. Schopf, M. Schott, J. F. P. Schouwenberg, J. Schovancova, S. Schramm, M. Schreyer, N. Schuh, A. Schulte, M. J. Schultens, H. -C. Schultz-Coulon, H. Schulz, M. Schumacher, B. A. Schumm, Ph. Schune, A. Schwartzman, T. A. Schwarz, H. Schweiger, Ph. Schwemling, R. Schwienhorst, J. Schwindling, T. Schwindt, G. Sciolla, F. Scuri, F. Scutti, J. Searcy, P. Seema, S. C. Seidel, A. Seiden, F. Seifert, J. M. Seixas, G. Sekhniaidze, K. Sekhon, S. J. Sekula, D. M. Seliverstov, N. Semprini-Cesari, C. Serfon, L. Serin, L. Serkin, M. Sessa, R. Seuster, H. Severini, T. Sfiligoj, F. Sforza, A. Sfyrla, E. Shabalina, N. W. Shaikh, L. Y. Shan, R. Shang, J. T. Shank, M. Shapiro, P. B. Shatalov, K. Shaw, S. M. Shaw, A. Shcherbakova, C. Y. Shehu, P. Sherwood, L. Shi, S. Shimizu, C. O. Shimmin, M. Shimojima, S. Shirabe, M. Shiyakova, A. Shmeleva, D. Shoaleh Saadi, M. J. Shochet, S. Shojaii, D. R. Shope, S. Shrestha, E. Shulga, M. A. Shupe, P. Sicho, A. M. Sickles, P. E. Sidebo, O. Sidiropoulou, D. Sidorov, A. Sidoti, F. Siegert, Dj. Sijacki, J. Silva, S. B. Silverstein, V. Simak, Lj. Simic, S. Simion, E. Simioni, B. Simmons, D. Simon, M. Simon, P. Sinervo, N. B. Sinev, M. Sioli, G. Siragusa, S. Yu. Sivoklokov, J. Sjölin, M. B. Skinner, H. P. Skottowe, P. Skubic, M. Slater, T. Slavicek, M. Slawinska, K. Sliwa, R. Slovak, V. Smakhtin, B. H. Smart, L. Smestad, J. Smiesko, S. Yu. Smirnov, Y. Smirnov, L. N. Smirnova, O. Smirnova, M. N. K. Smith, R. W. Smith, M. Smizanska, K. Smolek, A. A. Snesarev, I. M. Snyder, S. Snyder, R. Sobie, F. Socher, A. Soffer, D. A. Soh, G. Sokhrannyi, C. A. Solans Sanchez, M. Solar, E. Yu. Soldatov, U. Soldevila, A. A. Solodkov, A. Soloshenko, O. V. Solovyanov, V. Solovyev, P. Sommer, H. Son, H. Y. Song, A. Sood, A. Sopczak, V. Sopko, V. Sorin, D. Sosa, C. L. Sotiropoulou, R. Soualah, A. M. Soukharev, D. South, B. C. Sowden, S. Spagnolo, M. Spalla, M. Spangenberg, F. Spanò, D. Sperlich, F. Spettel, R. Spighi, G. Spigo, L. A. Spiller, M. Spousta, R. D. St. Denis, A. Stabile, R. Stamen, S. Stamm, E. Stanecka, R. W. Stanek, C. Stanescu, M. Stanescu-Bellu, M. M. Stanitzki, S. Stapnes, E. A. Starchenko, G. H. Stark, J. Stark, P. Staroba, P. Starovoitov, S. Stärz, R. Staszewski, P. Steinberg, B. Stelzer, H. J. Stelzer, O. Stelzer-Chilton, H. Stenzel, G. A. Stewart, J. A. Stillings, M. C. Stockton, M. Stoebe, G. Stoicea, P. Stolte, S. Stonjek, A. R. Stradling, A. Straessner, M. E. Stramaglia, J. Strandberg, S. Strandberg, A. Strandlie, M. Strauss, P. Strizenec, R. Ströhmer, D. M. Strom, R. Stroynowski, A. Strubig, S. A. Stucci, B. Stugu, N. A. Styles, D. Su, J. Su, S. Suchek, Y. Sugaya, M. Suk, V. V. Sulin, S. Sultansoy, T. Sumida, S. Sun, X. Sun, J. E. Sundermann, K. Suruliz, G. Susinno, M. R. Sutton, S. Suzuki, M. Svatos, M. Swiatlowski, I. Sykora, T. Sykora, D. Ta, C. Taccini, K. Tackmann, J. Taenzer, A. Taffard, R. Tafirout, N. Taiblum, H. Takai, R. Takashima, T. Takeshita, Y. Takubo, M. Talby, A. A. Talyshev, K. G. Tan, J. Tanaka, M. Tanaka, R. Tanaka, S. Tanaka, R. Tanioka, B. B. Tannenwald, S. Tapia Araya, S. Tapprogge, S. Tarem, G. F. Tartarelli, P. Tas, M. Tasevsky, T. Tashiro, E. Tassi, A. Tavares Delgado, Y. Tayalati, A. C. Taylor, G. N. Taylor, P. T. E. Taylor, W. Taylor, F. A. Teischinger, P. Teixeira-Dias, K. K. Temming, D. Temple, H. Ten Kate, P. K. Teng, J. J. Teoh, F. Tepel, S. Terada, K. Terashi, J. Terron, S. Terzo, M. Testa, R. J. Teuscher, T. Theveneaux-Pelzer, J. P. Thomas, J. Thomas-Wilsker, E. N. Thompson, P. D. Thompson, A. S. Thompson, L. A. Thomsen, E. Thomson, M. Thomson, M. J. Tibbetts, R. E. Ticse Torres, V. O. Tikhomirov, Yu. A. Tikhonov, S. Timoshenko, P. Tipton, S. Tisserant, K. Todome, T. Todorov, S. Todorova-Nova, J. Tojo, S. Tokár, K. Tokushuku, E. Tolley, L. Tomlinson, M. Tomoto, L. Tompkins, K. Toms, B. Tong, P. Tornambe, E. Torrence, H. Torres, E. Torró Pastor, J. Toth, F. Touchard, D. R. Tovey, T. Trefzger, A. Tricoli, I. M. Trigger, S. Trincaz-Duvoid, M. F. Tripiana, W. Trischuk, B. Trocmé, A. Trofymov, C. Troncon, M. Trottier-McDonald, M. Trovatelli, L. Truong, M. Trzebinski, A. Trzupek, J. C-L. Tseng, P. V. Tsiareshka, G. Tsipolitis, N. Tsirintanis, S. Tsiskaridze, V. Tsiskaridze, E. G. Tskhadadze, K. M. Tsui, I. I. Tsukerman, V. Tsulaia, S. Tsuno, D. Tsybychev, Y. Tu, A. Tudorache, V. Tudorache, A. N. Tuna, S. A. Tupputi, S. Turchikhin, D. Turecek, D. Turgeman, R. Turra, P. M. Tuts, M. Tyndel, G. Ucchielli, I. Ueda, M. Ughetto, F. Ukegawa, G. Unal, A. Undrus, G. Unel, F. C. Ungaro, Y. Unno, C. Unverdorben, J. Urban, P. Urquijo, P. Urrejola, G. Usai, L. Vacavant, V. Vacek, B. Vachon, C. Valderanis, E. Valdes Santurio, N. Valencic, S. Valentinetti, A. Valero, L. Valery, S. Valkar, J. A. Valls Ferrer, W. Van Den Wollenberg, P. C. Van Der Deijl, H. van der Graaf, N. van Eldik, P. van Gemmeren, J. Van Nieuwkoop, I. van Vulpen, M. C. van Woerden, M. Vanadia, W. Vandelli, R. Vanguri, A. Vaniachine, P. Vankov, G. Vardanyan, R. Vari, E. W. Varnes, T. Varol, D. Varouchas, A. Vartapetian, K. E. Varvell, J. G. Vasquez, G. A. Vasquez, F. Vazeille, T. Vazquez Schroeder, J. Veatch, V. Veeraraghavan, L. M. Veloce, F. Veloso, S. Veneziano, A. Ventura, M. Venturi, N. Venturi, A. Venturini, V. Vercesi, M. Verducci, W. Verkerke, J. C. Vermeulen, A. Vest, M. C. Vetterli, O. Viazlo, I. Vichou, T. Vickey, O. E. Vickey Boeriu, G. H. A. Viehhauser, S. Viel, L. Vigani, M. Villa, M. Villaplana Perez, E. Vilucchi, M. G. Vincter, V. B. Vinogradov, C. Vittori, I. Vivarelli, S. Vlachos, M. Vlasak, M. Vogel, P. Vokac, G. Volpi, M. Volpi, H. von der Schmitt, E. von Toerne, V. Vorobel, K. Vorobev, M. Vos, R. Voss, J. H. Vossebeld, N. Vranjes, M. Vranjes Milosavljevic, V. Vrba, M. Vreeswijk, R. Vuillermet, I. Vukotic, Z. Vykydal, P. Wagner, W. Wagner, H. Wahlberg, S. Wahrmund, J. Wakabayashi, J. Walder, R. Walker, W. Walkowiak, V. Wallangen, C. Wang, C. Wang, F. Wang, H. Wang, H. Wang, J. Wang, J. Wang, K. Wang, R. Wang, S. M. Wang, T. Wang, T. Wang, W. Wang, X. Wang, C. Wanotayaroj, A. Warburton, C. P. Ward, D. R. Wardrope, A. Washbrook, P. M. Watkins, A. T. Watson, M. F. Watson, G. Watts, S. Watts, B. M. Waugh, S. Webb, M. S. Weber, S. W. Weber, S. A. Weber, J. S. Webster, A. R. Weidberg, B. Weinert, J. Weingarten, C. Weiser, H. Weits, P. S. Wells, T. Wenaus, T. Wengler, S. Wenig, N. Wermes, M. Werner, M. D. Werner, P. Werner, M. Wessels, J. Wetter, K. Whalen, N. L. Whallon, A. M. Wharton, A. White, M. J. White, R. White, D. Whiteson, F. J. Wickens, W. Wiedenmann, M. Wielers, C. Wiglesworth, L. A. M. Wiik-Fuchs, A. Wildauer, F. Wilk, H. G. Wilkens, H. H. Williams, S. Williams, C. Willis, S. Willocq, J. A. Wilson, I. Wingerter-Seez, F. Winklmeier, O. J. Winston, B. T. Winter, M. Wittgen, J. Wittkowski, T. M. H. Wolf, M. W. Wolter, H. Wolters, S. D. Worm, B. K. Wosiek, J. Wotschack, M. J. Woudstra, K. W. Wozniak, M. Wu, M. Wu, S. L. Wu, X. Wu, Y. Wu, T. R. Wyatt, B. M. Wynne, S. Xella, D. Xu, L. Xu, B. Yabsley, S. Yacoob, D. Yamaguchi, Y. Yamaguchi, A. Yamamoto, S. Yamamoto, T. Yamanaka, K. Yamauchi, Y. Yamazaki, Z. Yan, H. Yang, H. Yang, Y. Yang, Z. Yang, W-M. Yao, Y. C. Yap, Y. Yasu, E. Yatsenko, K. H. Yau Wong, J. Ye, S. Ye, I. Yeletskikh, A. L. Yen, E. Yildirim, K. Yorita, R. Yoshida, K. Yoshihara, C. Young, C. J. S. Young, S. Youssef, D. R. Yu, J. Yu, J. M. Yu, J. Yu, L. Yuan, S. P. Y. Yuen, I. Yusuff, B. Zabinski, R. Zaidan, A. M. Zaitsev, N. Zakharchuk, J. Zalieckas, A. Zaman, S. Zambito, L. Zanello, D. Zanzi, C. Zeitnitz, M. Zeman, A. Zemla, J. C. Zeng, Q. Zeng, K. Zengel, O. Zenin, T. Ženiš, D. Zerwas, D. Zhang, F. Zhang, G. Zhang, H. Zhang, J. Zhang, L. Zhang, R. Zhang, R. Zhang, X. Zhang, Z. Zhang, X. Zhao, Y. Zhao, Z. Zhao, A. Zhemchugov, J. Zhong, B. Zhou, C. Zhou, L. Zhou, L. Zhou, M. Zhou, N. Zhou, C. G. Zhu, H. Zhu, J. Zhu, Y. Zhu, X. Zhuang, K. Zhukov, A. Zibell, D. Zieminska, N. I. Zimine, C. Zimmermann, S. Zimmermann, Z. Zinonos, M. Zinser, M. Ziolkowski, L. Živković, G. Zobernig, A. Zoccoli, M. zur Nedden, L. Zwalinski, ATLAS Collaboration

**Affiliations:** 1 Department of Physics, University of Adelaide, Adelaide, Australia; 2 Physics Department, SUNY Albany, Albany, NY USA; 3 Department of Physics, University of Alberta, Edmonton, AB Canada; 4 Department of Physics, Ankara University, Ankara, Turkey; 5 Istanbul Aydin University, Istanbul, Turkey; 6 Division of Physics, TOBB University of Economics and Technology, Ankara, Turkey; 7 LAPP, CNRS/IN2P3 and Université Savoie Mont Blanc, Annecy-le-Vieux, France; 8 High Energy Physics Division, Argonne National Laboratory, Argonne, IL USA; 9 Department of Physics, University of Arizona, Tucson, AZ USA; 10 Department of Physics, The University of Texas at Arlington, Arlington, TX USA; 11 Physics Department, University of Athens, Athens, Greece; 12 Physics Department, National Technical University of Athens, Zografou, Greece; 13 Department of Physics, The University of Texas at Austin, Austin, TX USA; 14 Institute of Physics, Azerbaijan Academy of Sciences, Baku, Azerbaijan; 15 Institut de Física d’Altes Energies (IFAE), The Barcelona Institute of Science and Technology, Barcelona, Spain; 16 Institute of Physics, University of Belgrade, Belgrade, Serbia; 17 Department for Physics and Technology, University of Bergen, Bergen, Norway; 18 Physics Division, Lawrence Berkeley National Laboratory and University of California, Berkeley, CA USA; 19 Department of Physics, Humboldt University, Berlin, Germany; 20 Albert Einstein Center for Fundamental Physics and Laboratory for High Energy Physics, University of Bern, Bern, Switzerland; 21 School of Physics and Astronomy, University of Birmingham, Birmingham, UK; 22 Department of Physics, Bogazici University, Istanbul, Turkey; 23 Department of Physics Engineering, Gaziantep University, Gaziantep, Turkey; 24 Faculty of Engineering and Natural Sciences, Istanbul Bilgi University, Istanbul, Turkey; 25 Faculty of Engineering and Natural Sciences, Bahcesehir University, Istanbul, Turkey; 26 Centro de Investigaciones, Universidad Antonio Narino, Bogotá, Colombia; 27 INFN Sezione di Bologna, Bologna, Italy; 28 Dipartimento di Fisica e Astronomia, Università di Bologna, Bologna, Italy; 29 Physikalisches Institut, University of Bonn, Bonn, Germany; 30 Department of Physics, Boston University, Boston, MA USA; 31 Department of Physics, Brandeis University, Waltham, MA USA; 32 Universidade Federal do Rio De Janeiro COPPE/EE/IF, Rio de Janeiro, Brazil; 33 Electrical Circuits Department, Federal University of Juiz de Fora (UFJF), Juiz de Fora, Brazil; 34 Federal University of Sao Joao del Rei (UFSJ), Sao Joao del Rei, Brazil; 35 Instituto de Fisica, Universidade de Sao Paulo, Sao Paulo, Brazil; 36 Physics Department, Brookhaven National Laboratory, Upton, NY USA; 37 Transilvania University of Brasov, Brasov, Romania; 38 National Institute of Physics and Nuclear Engineering, Bucharest, Romania; 39 Physics Department, National Institute for Research and Development of Isotopic and Molecular Technologies, Cluj Napoca, Romania; 40 University Politehnica Bucharest, Bucharest, Romania; 41 West University in Timisoara, Timisoara, Romania; 42 Departamento de Física, Universidad de Buenos Aires, Buenos Aires, Argentina; 43 Cavendish Laboratory, University of Cambridge, Cambridge, UK; 44 Department of Physics, Carleton University, Ottawa, ON Canada; 45 CERN, Geneva, Switzerland; 46 Enrico Fermi Institute, University of Chicago, Chicago, IL USA; 47 Departamento de Física, Pontificia Universidad Católica de Chile, Santiago, Chile; 48 Departamento de Física, Universidad Técnica Federico Santa María, Valparaiso, Chile; 49 Institute of High Energy Physics, Chinese Academy of Sciences, Beijing, China; 50 Department of Physics, Nanjing University, Jiangsu, China; 51 Physics Department, Tsinghua University, Beijing, 100084 China; 52 Laboratoire de Physique Corpusculaire, Clermont Université and Université Blaise Pascal and CNRS/IN2P3, Clermont-Ferrand, France; 53 Nevis Laboratory, Columbia University, Irvington, NY USA; 54 Niels Bohr Institute, University of Copenhagen, Kobenhavn, Denmark; 55 INFN Gruppo Collegato di Cosenza, Laboratori Nazionali di Frascati, Frascati, Italy; 56 Dipartimento di Fisica, Università della Calabria, Rende, Italy; 57 Faculty of Physics and Applied Computer Science, AGH University of Science and Technology, Kraków, Poland; 58 Marian Smoluchowski Institute of Physics, Jagiellonian University, Kraków, Poland; 59 Institute of Nuclear Physics, Polish Academy of Sciences, Kraków, Poland; 60 Physics Department, Southern Methodist University, Dallas, TX USA; 61 Physics Department, University of Texas at Dallas, Richardson, TX USA; 62 DESY, Hamburg and Zeuthen, Germany; 63 Lehrstuhl für Experimentelle Physik IV, Technische Universität Dortmund, Dortmund, Germany; 64 Institut für Kern- und Teilchenphysik, Technische Universität Dresden, Dresden, Germany; 65 Department of Physics, Duke University, Durham, NC USA; 66 SUPA-School of Physics and Astronomy, University of Edinburgh, Edinburgh, UK; 67 INFN Laboratori Nazionali di Frascati, Frascati, Italy; 68 Fakultät für Mathematik und Physik, Albert-Ludwigs-Universität, Freiburg, Germany; 69 Section de Physique, Université de Genève, Geneva, Switzerland; 70 INFN Sezione di Genova, Genoa, Italy; 71 Dipartimento di Fisica, Università di Genova, Genoa, Italy; 72 E. Andronikashvili Institute of Physics, Iv. Javakhishvili Tbilisi State University, Tbilisi, Georgia; 73 High Energy Physics Institute, Tbilisi State University, Tbilisi, Georgia; 74 II Physikalisches Institut, Justus-Liebig-Universität Giessen, Giessen, Germany; 75 SUPA-School of Physics and Astronomy, University of Glasgow, Glasgow, UK; 76 II Physikalisches Institut, Georg-August-Universität, Göttingen, Germany; 77 Laboratoire de Physique Subatomique et de Cosmologie, Université Grenoble-Alpes, CNRS/IN2P3, Grenoble, France; 78 Laboratory for Particle Physics and Cosmology, Harvard University, Cambridge, MA USA; 79 Department of Modern Physics, University of Science and Technology of China, Anhui, China; 80 Kirchhoff-Institut für Physik, Ruprecht-Karls-Universität Heidelberg, Heidelberg, Germany; 81 Physikalisches Institut, Ruprecht-Karls-Universität Heidelberg, Heidelberg, Germany; 82 ZITI Institut für technische Informatik, Ruprecht-Karls-Universität Heidelberg, Mannheim, Germany; 83 Faculty of Applied Information Science, Hiroshima Institute of Technology, Hiroshima, Japan; 84 Department of Physics, The Chinese University of Hong Kong, Shatin, NT Hong Kong; 85 Department of Physics, The University of Hong Kong, Pokfulam Road, Pokfulam, Hong Kong; 86 Department of Physics, The Hong Kong University of Science and Technology, Clear Water Bay, Kowloon, Hong Kong, China; 87 Department of Physics, Indiana University, Bloomington, IL USA; 88 Institut für Astro- und Teilchenphysik, Leopold-Franzens-Universität, Innsbruck, Austria; 89 University of Iowa, Iowa City, IA USA; 90 Department of Physics and Astronomy, Iowa State University, Ames, IA USA; 91 Joint Institute for Nuclear Research, JINR Dubna, Dubna, Russia; 92 KEK, High Energy Accelerator Research Organization, Tsukuba, Japan; 93 Graduate School of Science, Kobe University, Kobe, Japan; 94 Faculty of Science, Kyoto University, Kyoto, Japan; 95 Kyoto University of Education, Kyoto, Japan; 96 Department of Physics, Kyushu University, Fukuoka, Japan; 97 Instituto de Física La Plata, Universidad Nacional de La Plata and CONICET, La Plata, Argentina; 98 Physics Department, Lancaster University, Lancaster, UK; 99 INFN Sezione di Lecce, Lecce, Italy; 100 Dipartimento di Matematica e Fisica, Università del Salento, Lecce, Italy; 101 Oliver Lodge Laboratory, University of Liverpool, Liverpool, UK; 102 Department of Physics, Jožef Stefan Institute and University of Ljubljana, Ljubljana, Slovenia; 103 School of Physics and Astronomy, Queen Mary University of London, London, UK; 104 Department of Physics, Royal Holloway University of London, Surrey, UK; 105 Department of Physics and Astronomy, University College London, London, UK; 106 Louisiana Tech University, Ruston, LA USA; 107 Laboratoire de Physique Nucléaire et de Hautes Energies, UPMC and Université Paris-Diderot and CNRS/IN2P3, Paris, France; 108 Fysiska institutionen, Lunds universitet, Lund, Sweden; 109 Departamento de Fisica Teorica C-15, Universidad Autonoma de Madrid, Madrid, Spain; 110 Institut für Physik, Universität Mainz, Mainz, Germany; 111 School of Physics and Astronomy, University of Manchester, Manchester, UK; 112 CPPM, Aix-Marseille Université and CNRS/IN2P3, Marseille, France; 113 Department of Physics, University of Massachusetts, Amherst, MA USA; 114 Department of Physics, McGill University, Montreal, QC Canada; 115 School of Physics, University of Melbourne, Melbourne, VIC Australia; 116 Department of Physics, The University of Michigan, Ann Arbor, MI USA; 117 Department of Physics and Astronomy, Michigan State University, East Lansing, MI USA; 118 INFN Sezione di Milano, Milan, Italy; 119 Dipartimento di Fisica, Università di Milano, Milan, Italy; 120 B.I. Stepanov Institute of Physics, National Academy of Sciences of Belarus, Minsk, Republic of Belarus; 121 National Scientific and Educational Centre for Particle and High Energy Physics, Minsk, Republic of Belarus; 122 Group of Particle Physics, University of Montreal, Montreal, QC Canada; 123 P.N. Lebedev Physical Institute of the Russian Academy of Sciences, Moscow, Russia; 124 Institute for Theoretical and Experimental Physics (ITEP), Moscow, Russia; 125 National Research Nuclear University MEPhI, Moscow, Russia; 126 D.V. Skobeltsyn Institute of Nuclear Physics, M.V. Lomonosov Moscow State University, Moscow, Russia; 127 Fakultät für Physik, Ludwig-Maximilians-Universität München, Munich, Germany; 128 Max-Planck-Institut für Physik (Werner-Heisenberg-Institut), Munich, Germany; 129 Nagasaki Institute of Applied Science, Nagasaki, Japan; 130 Graduate School of Science and Kobayashi-Maskawa Institute, Nagoya University, Nagoya, Japan; 131 INFN Sezione di Napoli, Naples, Italy; 132 Dipartimento di Fisica, Università di Napoli, Naples, Italy; 133 Department of Physics and Astronomy, University of New Mexico, Albuquerque, NM USA; 134 Institute for Mathematics, Astrophysics and Particle Physics, Radboud University Nijmegen/Nikhef, Nijmegen, The Netherlands; 135 Nikhef National Institute for Subatomic Physics and University of Amsterdam, Amsterdam, The Netherlands; 136 Department of Physics, Northern Illinois University, DeKalb, IL USA; 137 Budker Institute of Nuclear Physics, SB RAS, Novosibirsk, Russia; 138 Department of Physics, New York University, New York, NY USA; 139 Ohio State University, Columbus, OH USA; 140 Faculty of Science, Okayama University, Okayama, Japan; 141 Homer L. Dodge Department of Physics and Astronomy, University of Oklahoma, Norman, OK USA; 142 Department of Physics, Oklahoma State University, Stillwater, OK USA; 143 Palacký University, RCPTM, Olomouc, Czech Republic; 144 Center for High Energy Physics, University of Oregon, Eugene, OR USA; 145 LAL, Univ. Paris-Sud, CNRS/IN2P3, Université Paris-Saclay, Orsay, France; 146 Graduate School of Science, Osaka University, Osaka, Japan; 147 Department of Physics, University of Oslo, Oslo, Norway; 148 Department of Physics, Oxford University, Oxford, UK; 149 INFN Sezione di Pavia, Pavia, Italy; 150 Dipartimento di Fisica, Università di Pavia, Pavia, Italy; 151 Department of Physics, University of Pennsylvania, Philadelphia, PA USA; 152 National Research Centre “Kurchatov Institute” B.P. Konstantinov Petersburg Nuclear Physics Institute, St. Petersburg, Russia; 153 INFN Sezione di Pisa, Pisa, Italy; 154 Dipartimento di Fisica E. Fermi, Università di Pisa, Pisa, Italy; 155 Department of Physics and Astronomy, University of Pittsburgh, Pittsburgh, PA USA; 156 Laboratório de Instrumentação e Física Experimental de Partículas-LIP, Lisbon, Portugal; 157 Faculdade de Ciências, Universidade de Lisboa, Lisbon, Portugal; 158 Department of Physics, University of Coimbra, Coimbra, Portugal; 159 Centro de Física Nuclear da Universidade de Lisboa, Lisbon, Portugal; 160 Departamento de Fisica, Universidade do Minho, Braga, Portugal; 161 Departamento de Fisica Teorica y del Cosmos and CAFPE, Universidad de Granada, Granada, Spain; 162 Dep Fisica and CEFITEC of Faculdade de Ciencias e Tecnologia, Universidade Nova de Lisboa, Caparica, Portugal; 163 Institute of Physics, Academy of Sciences of the Czech Republic, Prague, Czech Republic; 164 Czech Technical University in Prague, Prague, Czech Republic; 165 Faculty of Mathematics and Physics, Charles University in Prague, Prague, Czech Republic; 166 State Research Center Institute for High Energy Physics (Protvino), NRC KI, Moscow, Russia; 167 Particle Physics Department, Rutherford Appleton Laboratory, Didcot, UK; 168 INFN Sezione di Roma, Rome, Italy; 169 Dipartimento di Fisica, Sapienza Università di Roma, Rome, Italy; 170 INFN Sezione di Roma Tor Vergata, Rome, Italy; 171 Dipartimento di Fisica, Università di Roma Tor Vergata, Rome, Italy; 172 INFN Sezione di Roma Tre, Rome, Italy; 173 Dipartimento di Matematica e Fisica, Università Roma Tre, Rome, Italy; 174 Faculté des Sciences Ain Chock, Réseau Universitaire de Physique des Hautes Energies-Université Hassan II, Casablanca, Morocco; 175 Centre National de l’Energie des Sciences Techniques Nucleaires, Rabat, Morocco; 176 Faculté des Sciences Semlalia, Université Cadi Ayyad, LPHEA-Marrakech, Marrakech, Morocco; 177 Faculté des Sciences, Université Mohamed Premier and LPTPM, Oujda, Morocco; 178 Faculté des Sciences, Université Mohammed V, Rabat, Morocco; 179 DSM/IRFU (Institut de Recherches sur les Lois Fondamentales de l’Univers), CEA Saclay (Commissariat à l’Energie Atomique et aux Energies Alternatives), Gif-sur-Yvette, France; 180 Santa Cruz Institute for Particle Physics, University of California Santa Cruz, Santa Cruz, CA USA; 181 Department of Physics, University of Washington, Seattle, WA USA; 182 School of Physics, Shandong University, Shandong, China; 183 Department of Physics and Astronomy, Shanghai Key Laboratory for Particle Physics and Cosmology, Shanghai Jiao Tong University (also affiliated with PKU-CHEP), Shanghai, China; 184 Department of Physics and Astronomy, University of Sheffield, Sheffield, UK; 185 Department of Physics, Shinshu University, Nagano, Japan; 186 Fachbereich Physik, Universität Siegen, Siegen, Germany; 187 Department of Physics, Simon Fraser University, Burnaby, BC Canada; 188 SLAC National Accelerator Laboratory, Stanford, CA USA; 189 Faculty of Mathematics, Physics and Informatics, Comenius University, Bratislava, Slovakia; 190 Department of Subnuclear Physics, Institute of Experimental Physics of the Slovak Academy of Sciences, Kosice, Slovak Republic; 191 Department of Physics, University of Cape Town, Cape Town, South Africa; 192 Department of Physics, University of Johannesburg, Johannesburg, South Africa; 193 School of Physics, University of the Witwatersrand, Johannesburg, South Africa; 194 Department of Physics, Stockholm University, Stockholm, Sweden; 195 The Oskar Klein Centre, Stockholm, Sweden; 196 Physics Department, Royal Institute of Technology, Stockholm, Sweden; 197 Departments of Physics and Astronomy and Chemistry, Stony Brook University, Stony Brook, NY USA; 198 Department of Physics and Astronomy, University of Sussex, Brighton, UK; 199 School of Physics, University of Sydney, Sydney, Australia; 200 Institute of Physics, Academia Sinica, Taipei, Taiwan; 201 Department of Physics, Technion: Israel Institute of Technology, Haifa, Israel; 202 Raymond and Beverly Sackler School of Physics and Astronomy, Tel Aviv University, Tel Aviv, Israel; 203 Department of Physics, Aristotle University of Thessaloniki, Thessaloniki, Greece; 204 International Center for Elementary Particle Physics and Department of Physics, The University of Tokyo, Tokyo, Japan; 205 Graduate School of Science and Technology, Tokyo Metropolitan University, Tokyo, Japan; 206 Department of Physics, Tokyo Institute of Technology, Tokyo, Japan; 207 Tomsk State University, Tomsk, Russia; 208 Department of Physics, University of Toronto, Toronto, ON Canada; 209 INFN-TIFPA, Trento, Italy; 210 University of Trento, Trento, Italy; 211 TRIUMF, Vancouver, BC Canada; 212 Department of Physics and Astronomy, York University, Toronto, ON Canada; 213 Faculty of Pure and Applied Sciences, and Center for Integrated Research in Fundamental Science and Engineering, University of Tsukuba, Tsukuba, Japan; 214 Department of Physics and Astronomy, Tufts University, Medford, MA USA; 215 Department of Physics and Astronomy, University of California Irvine, Irvine, CA USA; 216 INFN Gruppo Collegato di Udine, Sezione di Trieste, Udine, Italy; 217 ICTP, Trieste, Italy; 218 Dipartimento di Chimica Fisica e Ambiente, Università di Udine, Udine, Italy; 219 Department of Physics and Astronomy, University of Uppsala, Uppsala, Sweden; 220 Department of Physics, University of Illinois, Urbana, IL USA; 221 Instituto de Fisica Corpuscular (IFIC) and Departamento de Fisica Atomica, Molecular y Nuclear and Departamento de Ingeniería Electrónica and Instituto de Microelectrónica de Barcelona (IMB-CNM), University of Valencia and CSIC, Valencia, Spain; 222 Department of Physics, University of British Columbia, Vancouver, BC Canada; 223 Department of Physics and Astronomy, University of Victoria, Victoria, BC Canada; 224 Department of Physics, University of Warwick, Coventry, UK; 225 Waseda University, Tokyo, Japan; 226 Department of Particle Physics, The Weizmann Institute of Science, Rehovot, Israel; 227 Department of Physics, University of Wisconsin, Madison, WI USA; 228 Fakultät für Physik und Astronomie, Julius-Maximilians-Universität, Würzburg, Germany; 229 Fakultät für Mathematik und Naturwissenschaften, Fachgruppe Physik, Bergische Universität Wuppertal, Wuppertal, Germany; 230 Department of Physics, Yale University, New Haven, CT USA; 231 Yerevan Physics Institute, Yerevan, Armenia; 232 Centre de Calcul de l’Institut National de Physique Nucléaire et de Physique des Particules (IN2P3), Villeurbanne, France; 233 CERN, 1211 Geneva 23, Switzerland

## Abstract

A search for supersymmetry in events with large missing transverse momentum, jets, and at least one hadronically decaying tau lepton has been performed using 3.2 fb$^{-1}$ of proton–proton collision data at $\sqrt{s}=13{\mathrm {\ TeV}}$ recorded by the ATLAS detector at the Large Hadron Collider in 2015. Two exclusive final states are considered, with either exactly one or at least two tau leptons. No excess over the Standard Model prediction is observed in the data. Results are interpreted in the context of gauge-mediated supersymmetry breaking and a simplified model of gluino pair production with tau-rich cascade decays, substantially improving on previous limits. In the GMSB model considered, supersymmetry-breaking scale ($\Lambda$) values below $92 {\mathrm {\ TeV}}$ are excluded at the 95% confidence level, corresponding to gluino masses below $2000 {\mathrm {\ GeV}}$. For large values of $\tan \beta$, values of $\Lambda$ up to $107 {\mathrm {\ TeV}}$ and gluino masses up to $2300 {\mathrm {\ GeV}}$ are excluded. In the simplified model, gluino masses are excluded up to $1570 {\mathrm {\ GeV}}$ for neutralino masses around $100 {\mathrm {\ GeV}}$. Neutralino masses below $700 {\mathrm {\ GeV}}$ are excluded for all gluino masses between 800 and $1500 {\mathrm {\ GeV}}$, while the strongest exclusion of $750 {\mathrm {\ GeV}}$ is achieved for gluino masses around $1450 {\mathrm {\ GeV}}$.

## Introduction

Supersymmetry (SUSY) [1–6] introduces a symmetry between fermions and bosons, resulting in a SUSY partner (sparticle) for each Standard Model (SM) particle, with identical mass and quantum numbers, and a difference of half a unit of spin. Squarks ($\tilde{q}$), gluinos ($\tilde{g}$), sleptons ($\tilde{\ell }$) and sneutrinos ($\tilde{\nu }$) are the superpartners of the quarks, gluons, charged leptons and neutrinos, respectively. The SUSY partners of the gauge and Higgs bosons are called gauginos and higgsinos, respectively. The charged electroweak gaugino and higgsino states mix to form charginos ($\tilde{\chi }_{i}^{\pm }$, $i = 1,2$), and the neutral states mix to form neutralinos ($\tilde{\chi }_{j}^{0}$, $j = 1,2,3,4$). Finally, the gravitino ($\tilde{G}$) is the SUSY partner of the graviton. As no supersymmetric particle has been observed, SUSY must be a broken symmetry. In general, the minimal supersymmetric Standard Model (MSSM) allows couplings which violate baryon- and lepton-number conservation, leading to, for example, a short proton lifetime. To ensure accordance with established measurements, *R*-parity [7–11] conservation is often assumed. In this scenario, sparticles are produced in pairs and decay through cascades involving SM particles and other sparticles until the lightest sparticle (LSP), which is stable, is produced.

Final states with tau leptons are of particular interest in SUSY searches, although they are experimentally challenging. Light sleptons could play a role in the co-annihilation of neutralinos in the early universe, and models with light scalar taus are consistent with dark-matter searches [12]. Furthermore, should SUSY or any other physics beyond the Standard Model (BSM) be discovered, independent studies of all three lepton flavours are necessary to investigate the coupling structure of the new physics, especially with regard to lepton universality. If squarks and gluinos are within the reach of the Large Hadron Collider (LHC), their production may be among the dominant SUSY processes.

This article reports on an inclusive search for squarks and gluinos produced via the strong interaction in events with jets, at least one hadronically decaying tau lepton, and large missing transverse momentum from undetected LSPs. Two distinct topologies are studied, with one tau lepton ($1\tau$) or two or more tau leptons ($2\tau$) in the final state. These mutually exclusive channels are optimised separately. The analysis is performed using 3.2 ${\mathrm{{fb}^{-1}}}$ of proton–proton (*pp*) collision data at $\sqrt{s}=13 {\mathrm {\ TeV}}$ recorded with the ATLAS detector at the LHC in 2015. Two SUSY scenarios are considered: a gauge-mediated SUSY-breaking (GMSB) model, and a simplified model of gluino pair production. Previous searches based on the same final state have been reported by the ATLAS [13, 14] and CMS [15] collaborations.

In GMSB models [16–18], SUSY breaking is communicated from a hidden sector to the visible sector by a set of messenger fields that share the gauge interactions of the SM. SUSY is spontaneously broken in the messenger sector, leading to massive, non-degenerate messenger fields. Gauginos acquire their masses via one-loop diagrams involving messengers. Squarks and sleptons, which do not couple directly to the messenger sector, get their masses from two-loop diagrams involving messengers and SM gauge bosons, or messengers and gauginos. The free parameters of GMSB models are the SUSY-breaking mass scale in the messenger sector ($\Lambda$), the messenger mass scale ($M_\mathrm {mes}$), the number of messenger multiplets ($N_5$) of the $\mathbf {5}+{\varvec{\bar{5}}}$ representation of $\text {SU}(5)$, the ratio of the two Higgs-doublet vacuum expectation values at the electroweak scale ($\tan \beta$), the sign of the Higgsino mass term in the superpotential ($\mathrm { sign}(\mu )=\pm 1$), and a gravitino-mass scale factor ($C_\mathrm {grav}$). Masses of gauginos and sfermions are proportional to $\Lambda$, and scale as $N_5$ and $\sqrt{N_5}$, respectively. The $M_\mathrm {mes}$ scale is required to be larger than $\Lambda$, to avoid tachyonic messengers and charge- and colour-breaking vacua, and lower than the Planck mass to suppress flavour violation. The latter condition implies that the lightest supersymmetric particle is a very light gravitino. The $C_\mathrm {grav}$ parameter, which results from the mechanism communicating SUSY breaking to the messengers, mainly affects the decay rate of the next-to-lightest supersymmetric particle (NLSP) into the LSP.

**Fig. 1:**
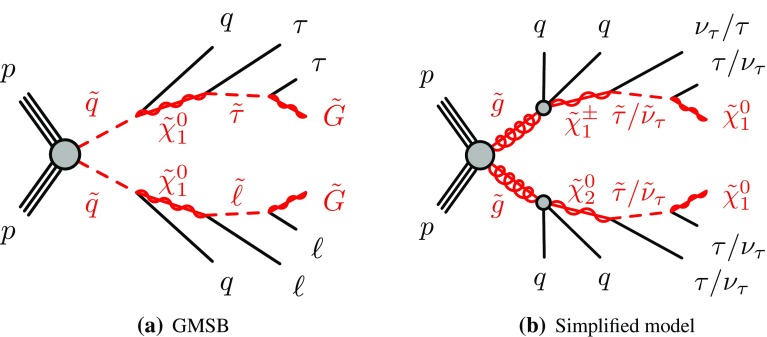
Diagrams illustrating the two SUSY scenarios studied in this analysis. In the GMSB model, the scalar lepton $\tilde{\ell }$ is preferentially a scalar tau $\tilde{\tau } _1$ for high values of $\tan \beta$

As in previous ATLAS searches [13, 14], the GMSB model is probed as a function of $\Lambda$ and $\tan \beta$, and the other parameters are set to $M_\mathrm {mes} =250$ TeV, $N_{\mathrm 5}=3$, $\mathrm {sign}(\mu )=1$ and $C_\mathrm {grav}=1$. For this choice of parameters, the NLSP is the lightest scalar tau ($\tilde{\tau }_1$) for large values of $\tan \beta$, while for lower $\tan \beta$ values, the $\tilde{\tau }_1$ and the superpartners of the right-handed electron and muon ($\tilde{e} _{\text {R}},\tilde{\mu } _{\text {R}}$) are almost degenerate in mass. The squark–antisquark production mechanism dominates at high values of $\Lambda$. A typical GMSB signal process is displayed in Fig. 1a. The value of $C_\mathrm {grav}$ corresponds to prompt decays of the NLSP. The region of small $\Lambda$ and large $\tan \beta$ is unphysical since it leads to tachyonic states.

The other signal model studied in this analysis is a simplified model of gluino pair production [19] in an *R*-parity-conserving scenario. It is inspired by generic models such as the phenomenological MSSM [20, 21] with dominant gluino pair production, light $\tilde{\tau }_1$ and a $\tilde{\chi }_1^0$ LSP. Gluinos are assumed to undergo a two-step cascade decay leading to tau-rich final states, as shown in Fig. 1b. The two free parameters of the model are the masses of the gluino ($m_{\tilde{g}}$) and the LSP ($m_{\tilde{\chi }_1^0}$). Assumptions are made about the masses of other sparticles, namely the $\tilde{\tau }_1$ and $\tilde{\nu }_\tau$ are mass-degenerate, and the $\tilde{\chi }_2^0$ and $\tilde{\chi }_1^\pm$ are also mass-degenerate, with

1 $$\begin{aligned} m_{\tilde{\chi }_1^\pm } = m_{\tilde{\chi }_2^0}= \frac{1}{2}(m_{\tilde{g}} + m_{\tilde{\chi }_1^0}), \quad m_{\tilde{\tau }_1}=m_{\tilde{\nu }_\tau }=\frac{1}{2}(m_{\tilde{\chi }_1^\pm } + m_{\tilde{\chi }_1^0}). \end{aligned}$$

Gluinos are assumed to decay to $\tilde{\chi }^{\pm }_{1} q \bar{q}'$ and $\tilde{\chi }^0_{2} q \bar{q}$ with equal branching ratios, where $q,q'$ denote generic first- and second-generation quarks. Neutralinos $\tilde{\chi }^0_{2}$ are assumed to decay to $\tilde{\tau } \tau$ and $\tilde{\nu }_{\tau } \nu _{\tau }$ with equal probability, and charginos $\tilde{\chi }^{\pm }_{1}$ are assumed to decay to $\tilde{\nu }_{\tau }\tau$ and $\tilde{\tau } \nu _{\tau }$ with equal probability. In the last step of the decay chain, $\tilde{\tau }$ and $\tilde{\nu }_{\tau }$ are assumed to decay to $\tau \tilde{\chi }^0_{1}$ and $\nu _\tau \tilde{\chi }^0_{1}$, respectively. All other SUSY particles are kinematically decoupled. The topology of signal events depends on the mass splitting between the gluino and the LSP. The sparticle decay widths are assumed to be small compared to sparticle masses, such that they play no role in the kinematics.

## The ATLAS detector

The ATLAS experiment is described in detail in Ref. [22]. It is a multi-purpose detector with a forward–backward symmetric cylindrical geometry and a solid angle^1^ coverage of nearly 4$\pi$.

The inner tracking detector (ID), covering the region $|\eta |<2.5$, consists of a silicon pixel detector, a silicon microstrip detector and a transition radiation tracker. The innermost layer of the pixel detector, the insertable B-layer [23], was installed between Run 1 and Run 2 of the LHC. The inner detector is surrounded by a thin superconducting solenoid providing a 2T magnetic field, and by a finely segmented lead/liquid-argon (LAr) electromagnetic calorimeter covering the region $|\eta |<3.2$. A steel/scintillator-tile hadronic calorimeter provides coverage in the central region $|\eta |<1.7$. The end-cap and forward regions, covering the pseudorapidity range $1.5<|\eta |<4.9$, are instrumented with electromagnetic and hadronic LAr calorimeters, with either steel, copper or tungsten as the absorber material.

A muon spectrometer system incorporating large superconducting toroidal air-core magnets surrounds the calorimeters. Three layers of precision wire chambers provide muon tracking coverage in the range $|\eta |<2.7$, while dedicated fast chambers are used for triggering in the region $|\eta |<2.4$.

The trigger system, composed of two stages, was upgraded [24] before Run 2. The Level-1 trigger system, implemented with custom hardware, uses information from calorimeters and muon chambers to reduce the event rate from 40 MHz to a maximum of 100 kHz. The second stage, called the High-Level Trigger (HLT), reduces the data acquisition rate to about 1 kHz. The HLT is based on software and runs reconstruction algorithms similar to those used in the offline reconstruction.

## Data and simulation samples

The data used in this analysis consist of *pp* collisions at a centre-of-mass energy of $\sqrt{s}=13$ TeV delivered by the LHC with a 25 ns bunch spacing and recorded by the ATLAS detector from August to November 2015. Data quality requirements are applied to ensure that all sub-detectors were operating normally, and that LHC beams were in stable-collision mode. The integrated luminosity of the resulting data set is $3.16 \pm 0.07$ ${\mathrm{{fb}^{-1}}}$.

Simulated Monte Carlo (MC) event samples are used to model both the SUSY signals and SM backgrounds, except multi-jet production, which is evaluated from data. All MC samples are generated at $\sqrt{s}=13$ TeV. In addition to the hard-scattering process, soft *pp* interactions (pile-up) are included in the simulation using the Pythia 8.186 [25] generator with the A2 [26] set of tuned parameters (tune) and MSTW2008LO [27] parton density function (PDF) set. Generated events are reweighted such that the average number of *pp* interactions per bunch crossing has the same distribution in data and simulation. For SM background samples, the interactions between generated particles and the detector material are simulated [28] using Geant4 [29] and a detailed description of the ATLAS detector. In the case of signal samples, a parameterised fast simulation [30] is used to describe the energy deposits in the calorimeters.

The *W*+jets and *Z*+jets processes are simulated with the Sherpa 2.1.1 [31] generator. Matrix elements (ME) are calculated for up to two partons at next-to-leading order (NLO) and up to four additional partons at leading order (LO) in perturbative QCD using the OpenLoops [32] and Comix [33] matrix element generators, respectively. The polarisation of tau leptons in $W(\tau \nu )$+jets and $Z(\tau \tau )$+jets events is handled by the TauSpinner [34] program. The phase-space merging between the Sherpa parton shower (PS) [35] and matrix elements follows the ME+PS@NLO prescription [36]. The CT10 [37] PDF set is used in conjunction with dedicated parton-shower tuning. Simulated samples are generated in bins of the transverse momentum ($p_{\text {T}}$) of the vector boson. The inclusive cross sections are normalised to a next-to-next-to-leading-order (NNLO) calculation [38] in perturbative QCD based on the FEWZ program [39].

For the simulation of $t\bar{t}$ and single-top-quark production in the *Wt*- and *s*-channels, the Powheg-Box v2 [40] generator is used with the CT10 PDF set for the matrix elements calculation. Electroweak *t*-channel single-top-quark events are generated using the Powheg-Box v1 generator. This generator uses the four-flavour scheme for the NLO matrix element calculation together with the fixed four-flavour CT10f4 PDF set. For all top quark processes, top quark spin correlations are taken into account (for *t*-channel production, top quarks are decayed using MadSpin [41]). The parton shower, hadronisation, and underlying event are simulated using Pythia 6.428 [42] with the CTEQ6L1 [43] PDF set and the corresponding Perugia 2012 tune [44]. Cross sections are calculated at NNLO in perturbative QCD with resummation of next-to-next-to-leading logarithmic (NNLL) soft gluon terms using the Top++ 2.0 program [45].

Diboson production is simulated using the Sherpa 2.1.1 generator with the CT10 PDF set. Processes with fully leptonic final states are calculated with up to one ($4\ell$, $2\ell +2\nu$) or no partons ($3\ell +1\nu$) at NLO and up to three additional partons at LO. Diboson processes with one of the bosons decaying hadronically and the other leptonically are simulated with up to 1 (*ZZ*) or 0 (*WW*, *WZ*) partons at NLO and up to 3 additional partons at LO. The generator cross sections are used for these samples.

The simplified-model signal samples are generated using MG5_aMC@NLO v2.2.3 [46] interfaced to Pythia 8.186 with the A14 tune [47] for the modelling of the parton shower, hadronisation and underlying event. The ME calculation is performed at tree level and includes the emission of up to two additional partons. The PDF set used for the generation is NNPDF23LO [48]. The ME–PS matching is done using the CKKW-L prescription [49], with a matching scale set to one quarter of the gluino mass. The GMSB signal samples are generated with the Herwig++ 2.7.1 [50] generator, with CTEQ6L1 PDFs and the UE-EE-5-CTEQ6L1 tune [51], using input files generated in the SLHA format with the SPheno v3.1.12 [52] program. The parton shower evolution is performed using an algorithm described in Refs. [50, 53–55]. Signal cross sections are calculated at NLO in the strong coupling constant, adding the resummation of soft gluon emission at next-to-leading-logarithm accuracy [56–60]. The nominal cross section and the uncertainty are taken from an envelope of cross-section predictions using different PDF sets and factorisation and renormalisation scales, as described in Ref. [61].

## Reconstruction of final-state objects

This analysis primarily requires the presence of jets, hadronically decaying tau leptons and missing transverse momentum in the final state. Jet *b*-tagging is used to separate the top quark background from vector bosons produced in association with jets (*V*+jets, where $V=W,Z$). Electrons and muons are vetoed in the $1\tau$ channel, and muons are explicitly used in the $2\tau$ channel for background modelling studies.

Primary vertices are reconstructed using inner-detector tracks with $p_{\text {T}} >400 {\mathrm {\ MeV}}$ that satisfy requirements on the number of hits in silicon tracking devices [62]. Primary vertex candidates are required to have at least two associated tracks, and the candidate with the largest $\sum p_{\text {T}} ^2$ is chosen as the primary vertex.

Jets are reconstructed with the anti-$k_t$ clustering algorithm [63] with a distance parameter $R=0.4$. Clusters of topologically connected calorimeter cells [64] with energy above the noise threshold, calibrated at the electromagnetic energy scale, are used as input. The jet energy is calibrated using a set of global sequential calibrations [65, 66]. Energy from pile-up interactions is subtracted based on the jet area and the median energy density computed for each event [67]. Jets are required to have $p_{\text {T}} > 20 {\mathrm {\ GeV}}$ and $|\eta | < 2.8$. The discrimination between hard-interaction jets and pile-up jets is achieved by a jet-vertex-tagging algorithm [68]. Events with jets originating from cosmic rays, beam background or detector noise are vetoed using the *loose* quality requirements defined in Ref. [69]. Jets containing *b*-hadrons (*b*-jets) are identified using a multivariate algorithm exploiting the long lifetime, high decay multiplicity, hard fragmentation, and large mass of *b*-hadrons [70]. The *b*-tagging algorithm identifies genuine *b*-jets with an efficiency of approximately 70% in simulated $t\bar{t}$ events. The rejection rates for *c*-jets, hadronically decaying tau leptons, and light-quark or gluon jets are approximately 8, 26 and 440, respectively [71].

Electron candidates are reconstructed from an isolated energy deposit in the electromagnetic calorimeter matched to an inner-detector track. They are required to have $p_{\text {T}} > 10 {\mathrm {\ GeV}}$, $|\eta | < 2.47$, and candidates reconstructed in the transition region between the barrel and end-cap calorimeters ($1.37<|\eta |<1.52$) are discarded. Electrons are required to satisfy a *loose* likelihood identification requirement [72, 73] based on calorimeter shower shapes and track properties. The significance of the transverse impact parameter of the electron track is required to be less than five.

Muon candidates are reconstructed in the region $|\eta | < 2.5$ from muon spectrometer tracks matching ID tracks. Muons are required to have $p_{\text {T}} > 10 {\mathrm {\ GeV}}$ and pass the *medium* identification requirements defined in Ref. [74], based on the number of hits in the ID and muon spectrometer, and the compatibility of the charge-to-momentum ratio measured in the two detector systems.

Hadronically decaying tau leptons are reconstructed [75] from anti-$k_t$ jets with $E_\mathrm {T}\ge 10{\mathrm {\ GeV}}$ and $|\eta |<2.5$ calibrated with a local cluster weighting technique [76]. Tau candidates are built from clusters of calorimeter cells within a cone of size $\Delta R \equiv \sqrt{\smash [b]{(\Delta \eta )^2+(\Delta \phi )^2}}=0.2$ centred on the jet axis. Tau energy scale corrections are applied to subtract the energy originating from pile-up interactions and correct for the calorimeter response. Tau leptons are required to have $p_{\text {T}} >20 {\mathrm {\ GeV}}$, and candidates reconstructed within the transition region $1.37< |\eta | <1.52$ are discarded. Tau leptons are required to have either one or three associated tracks, with a charge sum of $\pm 1$. A boosted-decision-tree discriminant is used to separate jets from tau leptons. It relies on track variables from the inner detector as well as longitudinal and transverse shower-shape variables from the calorimeters. The analysis makes use of *loose* and *medium* tau leptons, corresponding to identification working points with efficiencies of 60 and 55% for one-track tau leptons, respectively, and 50 and 40% for three-track tau leptons, respectively. Electrons mis-identified as 1-track tau leptons are rejected by imposing a $p_{\text {T}}$- and $|\eta |$-dependent requirement on the electron likelihood, which provides a constant efficiency of 95% for real tau leptons, with an inverse background efficiency (rejection factor) for electrons ranging from 30 to 150 depending on the $|\eta |$ region.

The missing transverse momentum vector $\vec {{p}}_\mathrm {T}^\mathrm {~miss}$, whose magnitude is denoted by $E_{\mathrm {T}}^{\mathrm {miss}}$, is defined as the negative vector sum of the transverse momenta of all identified physics objects (electrons, muons, jets, and tau leptons) and an additional soft-term. Therefore, the $E_{\mathrm {T}}^{\mathrm {miss}}$ calculation benefits from the dedicated calibration for each final-state object. The soft-term is constructed from all the tracks with $p_{\text {T}} >400{\mathrm {\ MeV}}$ originating from the primary vertex which are not associated with any physics object. This track-based definition makes the soft-term largely insensitive to pile-up [77].

After object reconstruction, an *overlap-removal* procedure is applied to remove ambiguities in case the same object is reconstructed by several algorithms. Tau candidates are discarded if they are found to overlap with a light lepton (electron or muon). If an electron and a muon are reconstructed using the same inner-detector track, the electron is discarded. For overlapping jets and electrons, the electron is kept. Light leptons in the vicinity of a jet are considered to originate from secondary decays within the jet and are discarded. Finally, in case a jet is reconstructed as a tau lepton, the tau candidate is retained. The successive steps of this procedure are summarised in Table 1. The final-state objects considered in the analysis are those surviving the overlap-removal algorithm.

**Table 1:** Overview of the successive steps in the overlap removal algorithm

	Object discarded	Object kept	Matching condition
1.	Loose tau	Electron	$\Delta R < 0.2$
2.	Loose tau	Muon	$\Delta R < 0.2$
3.	Electron	Muon	Shared inner-detector track
4.	Jet	Electron	$\Delta R < 0.2$
5.	Electron	Jet	$\Delta R < 0.4$
6.	Muon	Jet	$\Delta R < 0.4$
7.	Jet	Loose tau	$\Delta R < 0.2$

## Event selection

The trigger used in this analysis is the lowest-threshold missing transverse momentum trigger that was active without restrictions during the whole 2015 data-taking period. The efficiency of that trigger is measured using data collected by a set of single-jet triggers, with events containing at least two jets and one loose tau lepton. The trigger is found to have an efficiency greater than 99% when requiring $E_{\mathrm {T}}^{\mathrm {miss}} > 180 {\mathrm {\ GeV}}$ and a jet with $p_{\text {T}} > 120 {\mathrm {\ GeV}}$ in the offline selection, which is referred to as *trigger plateau* conditions.

After trigger requirements, a pre-selection common to both channels is applied to ensure that only well-reconstructed events enter the analysis. Events containing no reconstructed primary vertex, mis-measured muon tracks, cosmic muon candidates, jets originating from calorimeter noise or reconstructed near inactive areas in the calorimeter, are vetoed. The presence of a second jet with $p_{\text {T}} > 20 {\mathrm {\ GeV}}$ is required. To suppress the contribution from multi-jet events where large $E_{\mathrm {T}}^{\mathrm {miss}}$ would arise from jet energy mis-measurement, a minimum angular separation in the transverse plane is imposed between either of the two leading jets and the missing transverse momentum, $\Delta \phi (\text {jet}_{1,2},\vec {{p}}_\mathrm {T}^\mathrm {~miss})>0.4$.

As part of the pre-selection in the $1\tau$ channel, events are required to contain exactly one medium tau lepton and no light lepton. The veto against electrons and muons was used in Run 1 to ensure the $1\tau$ channel did not overlap with the $e\tau$ and $\mu \tau$ channels, which are not part of the present analysis. The lepton veto does not affect the expected sensitivity to the simplified model, and has been kept in the $1\tau$ channel. Events with two or more loose tau leptons are rejected to make the $1\tau$ and $2\tau$ channels statistically independent. In the $2\tau$ channel, at least two loose tau leptons are required at pre-selection level. No veto is applied against light leptons, as this would cause a sizeable selection inefficiency for GMSB signals.

In each channel, signal regions (SRs) are defined for several signal scenarios. The following kinematic variables are found to provide discrimination between signal and background, or between backgrounds themselves:

- the transverse mass of the system formed by $\vec {{p}}_\mathrm {T}^\mathrm {~miss}$ and a lepton $\ell$ assumed to be massless,
  2 $$\begin{aligned} m^\ell _{\mathrm {T}} \equiv m_\mathrm {T}(\ell ,\vec {{p}}_\mathrm {T}^\mathrm {~miss})=\sqrt{2p_{\text {T}} ^{\ell }E_{\mathrm {T}}^{\mathrm {miss}} (1-\cos \Delta \phi (\ell ,\vec {{p}}_\mathrm {T}^\mathrm {~miss}))}~. \end{aligned}$$
  For events where the missing transverse momentum and the lepton originate from a $W\rightarrow \ell \nu$ decay, the $m^\ell _{\mathrm {T}}$ distribution exhibits a Jacobian peak at the *W* boson mass. In the $1\tau$ channel, the $m_{\mathrm {T}} ^\tau$ variable is used. In the $2\tau$ channel, the $m_{\mathrm T}^{\tau _1}+m_{\mathrm T}^{\tau _2}$ variable based on the two leading tau leptons is used, and $m_\mathrm {T}^\mu$ is used for specific selections requiring a muon;
- the scalar sum of the transverse momenta of all tau leptons and jets in the event, $H_{\mathrm {T}} =\sum \limits _i p_{\text {T}} ^{\mathrm {\tau }_i}+ \sum \limits _j p_{\text {T}} ^{\mathrm {jet}_j}$ ;
- the magnitude of the missing transverse momentum, $E_{\mathrm {T}}^{\mathrm {miss}}$;
- the effective mass, $m_{\mathrm {eff}} =H_{\mathrm {T}} +E_{\mathrm {T}}^{\mathrm {miss}}$;
- the $m_\text {T2} ^{\tau \tau }$ variable [78, 79], also called *stransverse mass*, computed as
  3 $$\begin{aligned} m_\text {T2} ^{\tau \tau } =\sqrt{ \min \limits _{{\vec {p}_\mathrm {T}^{\,a}+ \vec {p}_\mathrm {T}^{\, b} = \vec {{p}}_\mathrm {T}^\mathrm {~miss}}}(\max [m_\mathrm {T}^2(\tau _1,\vec {p}_\mathrm {T}^{~a}) , m_\mathrm {T}^2(\tau _2,\vec {p}_\mathrm {T}^{~b})])}, \end{aligned}$$
  where (*a*, *b*) refers to two invisible particles that are assumed to be produced with transverse momentum vectors $\vec {p}_\mathrm {T}^{~a,b}$. In this calculation, (*a*, *b*) are assumed to be massless. The $m_\text {T2} ^{\tau \tau }$ distribution has a kinematic endpoint for processes where massive particles are pair-produced, each particle decaying to a tau lepton and an undetected particle. In cases where multiple tau leptons are produced in a decay chain, there is no a-priori way to select the pair leading to the desired characteristic. For events with more than two tau-lepton candidates, $m_\text {T2} ^{\tau \tau }$ is hence calculated using all possible tau-lepton pairs and the largest value is chosen;
- the sum of the transverse masses of all jets and of the two leading tau leptons in the event, $m_{\mathrm {T}} ^\mathrm {sum} = m_{\mathrm T}^{\tau _1}+m_{\mathrm T}^{\tau _2} + \sum \limits _i m_{\mathrm T}^{\mathrm {jet}_i}$ , where $m_{\mathrm T}^{\mathrm {jet}}$ is defined analogously to $m^\ell _{\mathrm {T}}$ ;
- the total number of jets, $N_\text {jet}$;
- the number of *b*-tagged jets, $N_{b\text {-jet}}$.

These variables are also used for the selection of control regions (CRs) and validation regions (VRs) in the context of background modelling studies, as discussed in Sect. 6.

**Fig. 2:**
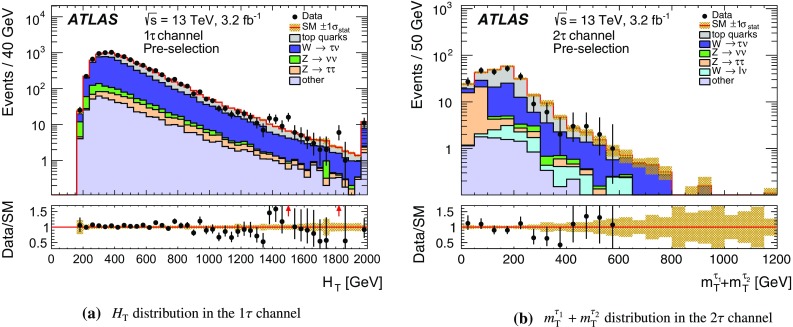
Kinematic distributions at pre-selection level, for the $1\tau$ and $2\tau$ channels. The last bin includes overflow events. The *shaded bands* indicate the statistical uncertainties in the background predictions. The contribution labelled as “other” includes diboson and multi-jet events, and the *V*+jets processes not explicitly listed in the legend. *Red arrows* in the data/SM ratio indicate bins where the corresponding entry falls outside the plotted range

Figure 2 shows example kinematic distributions at pre-selection level. The dominant backgrounds are $W(\tau \nu )$+jets and $t\bar{t}$ production in the $1\tau$ channel, with subdominant contributions from $Z(\nu \nu )$+jets and $Z(\tau \tau )$+jets. In the $2\tau$ channel, the pre-selection is dominated by $t\bar{t}$, $W(\tau \nu )$+jets, and $Z(\tau \tau )$+jets events. The multi-jet and diboson backgrounds respectively contribute to about 0.2 and 2% of the total background in the $1\tau$ channel, while their contribution in the $2\tau$ channel amounts to 1 and 5%, respectively.

In the $1\tau$ channel, three characteristic regions in the $(m_{\tilde{g}},m_{\tilde{\chi }_1^0})$ parameter space of the simplified model are chosen as benchmark scenarios for the SR optimisation, with small ($<100{\mathrm {\ GeV}}$), medium (500–$900{\mathrm {\ GeV}}$) and large ($>1200 {\mathrm {\ GeV}}$) mass splittings between the gluino and the LSP. In the following, the associated SRs are called Compressed, Medium-Mass and High-Mass SRs, respectively. The Compressed SR exploits topologies where a high-$p_{\text {T}}$ jet from initial-state radiation (ISR) recoils against the pair of gluinos. In this situation, the soft visible particles produced in the gluino decay receive a transverse Lorentz boost. While tau leptons and jets from gluino decays typically have low $p_{\text {T}}$, such events have substantial $E_{\mathrm {T}}^{\mathrm {miss}}$ since both LSPs tend to be emitted oppositely to the ISR jet in the transverse plane. In the case of large mass splitting, high-$p_{\text {T}}$ jets come mainly from gluino decays, and a high-$p_{\text {T}}$ requirement on the first two leading jets is effective in rejecting background without inducing a large inefficiency for the signal. A requirement on the transverse mass $m_{\mathrm {T}} ^\tau$ is applied in the Medium-Mass and High-Mass SRs to suppress $W(\tau \nu )$+jets events as well as semileptonic $t\bar{t}$ events with a tau lepton in the final state. The $H_{\mathrm {T}}$ variable is also used in these two SRs, as $H_{\mathrm {T}}$ increases for signal events with increasing mass splittings. No SR is defined for the GMSB model, as the expected sensitivity in the $1\tau$ channel is significantly lower than that in the $2\tau$ channel.

In the $2\tau$ channel, two SRs are defined for the simplified model to cover small ($<900{\mathrm {\ GeV}}$) and large ($>1200 {\mathrm {\ GeV}}$) mass-splitting scenarios. The Compressed SR imposes a requirement on $m_\text {T2} ^{\tau \tau }$ to exploit the fact that most of SM background contributions exhibit a kinematic endpoint around the *W* or *Z* boson mass, which is not the case for tau leptons produced in the cascade decay of gluinos. A requirement is also applied on $m_{\mathrm {T}} ^\mathrm {sum}$ to take advantage of the large $E_{\mathrm {T}}^{\mathrm {miss}}$ and the large jet and tau lepton multiplicity that is expected for signal events. The High-Mass SR includes a requirement on $H_{\mathrm {T}}$, which is efficient for High-Mass gluino signals. A requirement on $m_{\mathrm T}^{\tau _1}+m_{\mathrm T}^{\tau _2}$ is also applied. In $Z(\tau \tau )$+jets events, where $E_{\mathrm {T}}^{\mathrm {miss}}$ originates from neutrinos from tau lepton decays, the trigger plateau requirement selects events with a high-$p_{\text {T}}$ *Z* boson recoiling against jets in the transverse plane. This topology leads to tau leptons with a small $\Delta \phi$ separation, which results in low values of $m_{\mathrm T}^{\tau _1}+m_{\mathrm T}^{\tau _2}$ given that tau neutrinos are themselves collimated with the visible decay products of tau leptons. For dileptonic $t\bar{t}$ events with two tau leptons and large genuine $E_{\mathrm {T}}^{\mathrm {miss}}$, and for $W(\tau \nu )$+jets events and semileptonic $t\bar{t}$ events with a high-$p_{\text {T}}$ jet mis-identified as a tau lepton, $m_{\mathrm T}^{\tau _1}+m_{\mathrm T}^{\tau _2}$ can be larger, but even larger values are expected for High-Mass gluino signals. A signal region is also defined for the GMSB model, and targets more specifically squark–antisquark production rather than gluino pair production in the region $\Lambda \gtrsim 80 {\mathrm {\ TeV}}$, not excluded by Run 1 searches. Among the distinctive features that give large discrimination power to $H_{\mathrm {T}}$, decay chains are potentially longer than in the simplified model, and the almost-massless gravitino LSP leaves more phase space to other particles in the decay. Table 2 summarises the selection criteria for all the SRs of the $1\tau$ and $2\tau$ channels.

**Table 2:** Selection criteria for the signal regions (SRs) of the $1\tau$ channel (top) and the $2\tau$ channel (bottom)

$1\tau$ channel	Compressed SR	Medium-Mass SR	High-Mass SR
Trigger plateau	$E_{\mathrm {T}}^{\mathrm {miss}} > 180 {\mathrm {\ GeV}}$, $p_{\text {T}} ^\mathrm {jet_1} > 120 {\mathrm {\ GeV}}$		
Tau leptons	$N_\tau ^{\text {loose}} = N_\tau ^{\text {medium}}=1$, $p_{\text {T}} ^\tau >20 {\mathrm {\ GeV}}$		
Light leptons	$N_\ell =0\qquad$		
Multi-jet rejection	$\Delta \phi ({\text {jet}}_{1,2},\vec {{p}}_\mathrm {T}^\mathrm {~miss})\ge 0.4$		
$p_{\text {T}} ^{\tau }$	$< 45 {\mathrm {\ GeV}}$	–	–
$p_{\text {T}} ^{\mathrm{jet}_1}$	$> 300 {\mathrm {\ GeV}}$	–	$> 220 {\mathrm {\ GeV}}$
$p_{\text {T}} ^{\mathrm{jet}_2}$	–	–	$> 220 {\mathrm {\ GeV}}$
$N_\text {jet}$	$\ge 2$	$\ge 5$	$\ge 5$
$m_{\mathrm {T}} ^\tau$	$> 80 {\mathrm {\ GeV}}$	$> 200 {\mathrm {\ GeV}}$	$> 200 {\mathrm {\ GeV}}$
$E_{\mathrm {T}}^{\mathrm {miss}}$	$> 300 {\mathrm {\ GeV}}$	$> 300 {\mathrm {\ GeV}}$	–
$H_{\mathrm {T}}$	–	$> 550 {\mathrm {\ GeV}}$	$> 550 {\mathrm {\ GeV}}$
$2\tau$ channel	Compressed SR	High-Mass SR	GMSB SR
Trigger plateau	$E_{\mathrm {T}}^{\mathrm {miss}} > 180 {\mathrm {\ GeV}}$, $p_{\text {T}} ^{\mathrm{jet}_1} > 120 {\mathrm {\ GeV}}$		
Tau leptons	$N_\tau ^\mathrm{loose}\ge 2$, $p_{\text {T}} ^\tau >20 {\mathrm {\ GeV}}$		
Multi-jet rejection	$\Delta \phi (\mathrm{jet}_{1,2},\vec {{p}}_\mathrm {T}^\mathrm {~miss})\ge 0.4$		
$m_{\mathrm T}^{\tau _1}+m_{\mathrm T}^{\tau _2}$	–	$> 350 {\mathrm {\ GeV}}$	$> 150 {\mathrm {\ GeV}}$
$H_{\mathrm {T}}$	–	$> 800 {\mathrm {\ GeV}}$	$> 1700 {\mathrm {\ GeV}}$
$N_\text {jet}$	$\ge 2$	$\ge 3$	$\ge 2$
$m_\text {T2} ^{\tau \tau }$	$> 60 {\mathrm {\ GeV}}$	–	–
$m_{\mathrm {T}} ^\mathrm {sum}$	$> 1400 {\mathrm {\ GeV}}$	–	–

## Background estimation

To predict the background contributions in the SRs, the normalisation of the dominant backgrounds is fitted to data in dedicated CRs. In each channel and for each SR, a simultaneous fit over the relevant CRs is performed using HistFitter [80] to extract these normalisation factors. Control regions are designed to have an enhanced contribution from a single background process, with contributions from other backgrounds as small as possible to reduce the uncertainties originating from correlations between CRs. Furthermore, CRs are defined in phase-space regions close to that of SRs, to avoid the extrapolation of the background normalisations over very different kinematic regimes.

A set of VRs is defined in intermediate phase-space regions between a SR and its associated CRs, where signal contributions are small. These VRs are not part of the fit; they are used to compare the fitted background predictions with the observed data in the vicinity of the SRs before unblinding those.

### Vector-boson and top quark backgrounds

In both channels, the dominant backgrounds originate from SM processes involving the top quark or a massive vector boson and jets. These two backgrounds can be separated from each other by either requiring or vetoing the presence of a *b*-tagged jet in the event. In addition, a tau-lepton candidate can be either a genuine tau lepton (*true tau lepton*) from a $W(\tau \nu )$ decay or a jet mis-identified as a tau lepton (*fake tau lepton*), which leads to two types of CRs.

In CRs targeting true tau-lepton contributions, the normalisation factor is used to absorb the theoretical uncertainties in cross-section computations, the experimental uncertainties in the integrated luminosity, and potential differences in the tau-lepton reconstruction and identification efficiencies between data and simulation. In the case of fake tau-lepton contributions, the normalisation factor combines several effects: the quark/gluon composition of jets mis-identified as tau leptons, which is process-dependent, the parton shower and hadronisation models of the generator, and the modelling in the simulation of jet shower shapes in the calorimeter, which mainly depends on the Geant4 hadronic interaction model and the modelling of the ATLAS detector. Other contributions affecting the background normalisation include the modelling of the kinematics and acceptance of background processes. These contributions are absorbed into the true and fake tau-lepton normalisation factors in the $1\tau$ channel, while they are treated as a separate *kinematic* normalisation factor in the $2\tau$ channel to avoid double-counting (true- or fake-tau normalisation factors are applied to each tau lepton, while the kinematic normalisation factor is applied once per event).

**Fig. 3:**
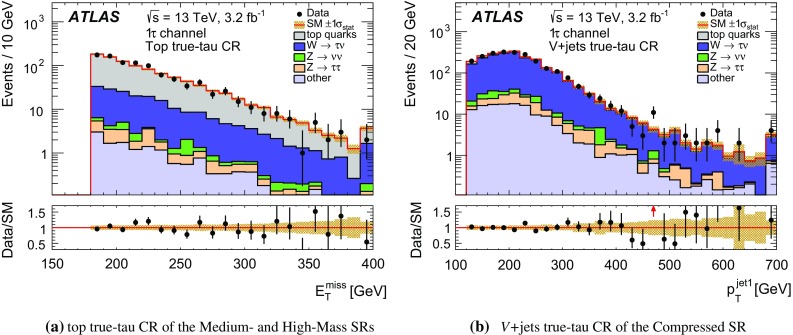
Missing transverse momentum and leading-jet $p_{\text {T}}$ distributions in two different control regions of the $1\tau$ channel after the fit, illustrating the overall background modelling in the CRs. By construction, the total fitted background is equal to the number of observed events in each CR. The last bin includes overflow events. The *shaded bands* indicate the statistical uncertainties in the background predictions. *Red arrows* in the data/SM ratio indicate bins where the corresponding entry falls outside the plotted range

In the $1\tau$ channel, four CRs are defined, with four associated normalisation factors. They target the top quark background (including $t\bar{t}$ and single-top-quark processes) with either a true or a fake tau lepton, and *V*+jets events with either a true or a fake tau lepton, respectively dominated by $W(\tau \nu )$+jets and $Z(\nu \nu )$+jets processes. The discrimination between true and fake tau-lepton contributions is achieved by a requirement on $m_\mathrm {T}^\tau$. A common set of four CRs is defined for the Medium-Mass and High-Mass SRs, due to the similarity of background compositions and event kinematics. These are separated from the SRs by an upper bound on $m_{\mathrm {T}} ^\tau$. Another set of four CRs is defined for the Compressed SR, to study more specifically the background modelling for low-$p_{\text {T}}$ tau leptons. These CRs are separated from the SR by an upper bound on $E_{\mathrm {T}}^{\mathrm {miss}}$. The selection criteria defining these CRs are summarised in Tables 3 and 4. Figure 3 illustrates the good modelling of the background in the various CRs after the fit.

Three types of VRs are used in the $1\tau$ channel for the Medium- and High-Mass SRs, to validate the background extrapolation from low-$H_{\mathrm {T}}$ to high-$H_{\mathrm {T}}$ for selections based on true tau leptons, and the extrapolations along $H_{\mathrm {T}}$ and $m_{\mathrm {T}} ^\tau$ for selections based on fake tau leptons. The separation of the VRs from the SRs is achieved by inverting the selections on $m_{\mathrm {T}} ^\tau$ or $H_{\mathrm {T}}$. For the Compressed SR, four VRs are used to validate the extrapolation of *V*+jets and top quark background predictions along $E_{\mathrm {T}}^{\mathrm {miss}}$, for both the true and fake tau-lepton selections. The separation of the VRs from the SRs is achieved by an inverted requirement on $m_{\mathrm {T}} ^\tau$ and $E_{\mathrm {T}}^{\mathrm {miss}}$, for true and fake tau-lepton VRs, respectively. The selection criteria defining all the VRs in the $1\tau$ channel are listed in Tables 3 and 4.

**Table 3:** Selection criteria defining the control and validation regions of the Compressed SR in the $1\tau$ channel. The pre-selection criteria are also applied although not shown in the table. Unless mentioned, criteria apply to both the top quark and *V*+jets background regions. Symbols* indicate that criteria are only applied to regions targeting the top quark background, and symbols$^{\dag }$ denote criteria only applied to *V*+jets background regions

$1\tau$ channel	True-tau CR	True-tau VR	Fake-tau CR	Fake-tau VR
$E_{\mathrm {T}}^{\mathrm {miss}}$	$180<E_{\mathrm {T}}^{\mathrm {miss}} <300 {\mathrm {\ GeV}}$	$>300 {\mathrm {\ GeV}}$	$180<E_{\mathrm {T}}^{\mathrm {miss}} <250 {\mathrm {\ GeV}}$	$250<E_{\mathrm {T}}^{\mathrm {miss}} <300 {\mathrm {\ GeV}}$
$m_{\mathrm T}^\tau$	$<100 {\mathrm {\ GeV}}$	$<80 {\mathrm {\ GeV}}$	$100<m_{\mathrm T}^\tau <330 {\mathrm {\ GeV}}$	$100<m_{\mathrm T}^\tau <330 {\mathrm {\ GeV}}$
$H_{\mathrm T}$	–	$>400 {\mathrm {\ GeV}}$	$<550^{{*}} / 400 ^{{\dag }} {\mathrm {\ GeV}}$	$>550^{{*}} / 400^{{\dag }} {\mathrm {\ GeV}}$
$p_{\text {T}} ^\tau$	$<45 {\mathrm {\ GeV}}$	$< 60{\mathrm {\ GeV}}$	$< 45{\mathrm {\ GeV}}$	$< 60{\mathrm {\ GeV}}$
$N_{\mathrm {jet}}$	–	$\ge 4^{{*}}$	–	$\ge 4^{{*}}$
$N_{b-\mathrm {jet}}$	$\ge 1^{{*}} / = 0^{{\dag }}$	$\ge 1^{{*}} / = 0^{{\dag }}$	$\ge 1^{{*}} / = 0^{{\dag }}$	$\ge 1^{{*}} / = 0^{\dag }$
$\Delta \phi (\mathrm {jet_1},\vec {{p}}_\mathrm {T}^\mathrm {~miss})$	$>1.8^{\dag }$	–	$>2.0 ^{{*}}$	–
$\Delta \phi (\tau ,\vec {{p}}_\mathrm {T}^\mathrm {~miss})$	–	–	$>1.0$	$>1.0$
$E_{\mathrm {T}}^{\mathrm {miss}}/m_{\mathrm {eff}}$	–	–	$>0.2^{{*}} / 0.3^{{\dag }}$	$>0.2^{{*}} / 0.3^{{\dag }}$

**Table 4:** Selection criteria defining the control and validation regions of the Medium-Mass and High-Mass SRs in the $1\tau$ channel. The pre-selection criteria are also applied although not shown in the table. Unless mentioned, criteria apply to both the top quark and *V*+jets background regions. Symbols* indicate that criteria are only applied to regions targeting the top quark background, and symbols$^{\dag }$ denote criteria only applied to *V*+jets background regions

$1\tau$ channel	True-tau CR	True-tau VR	Fake-tau CR	Fake-tau VR1	Fake-tau VR2
$E_{\mathrm {T}}^{\mathrm {miss}}$	–	–	–	$< 400^{{\dag }} {\mathrm {\ GeV}}$	–
$m_{\mathrm T}$	$<100 {\mathrm {\ GeV}}$	$<100 {\mathrm {\ GeV}}$	$100<m_{\mathrm T}<200 {\mathrm {\ GeV}}$	$100<m_{\mathrm T}<200 {\mathrm {\ GeV}}$	$200<m_{\mathrm T}<330 {\mathrm {\ GeV}}$
$H_{\mathrm T}$	$<550 {\mathrm {\ GeV}}$	$>550 {\mathrm {\ GeV}}$	$<550^{{*}}/ 400^{{\dag }} {\mathrm {\ GeV}}$	$>550^{{*}} {\mathrm {\ GeV}}$	$<550 {\mathrm {\ GeV}}$
	–	–	–	$400< H_\mathrm {T}< 700^{{\dag }} {\mathrm {\ GeV}}$	–
$p_{\text {T}} ^\tau$	–	$> 45{\mathrm {\ GeV}}$	–	–	–
$N_{\mathrm {jet}}$	$\ge 4$	$\ge 4$	–	$\ge 4^{{*}}$	$\ge 4^{{*}}$
$N_{b{\text {-}}\mathrm {jet}}$	$\ge 1^{{*}} / = 0^{{\dag }}$	$\ge 1^{{*}} / = 0^{{\dag }}$	$\ge 1^{{*}} / = 0^{{\dag }}$	$\ge 1^{{*}} / = 0^{{\dag }}$	$\ge 1^{{*}} / = 0^{{\dag }}$
$p_{\text {T}} ^\mathrm {jet_1}$	–	–	–	$< 500^{{\dag }} {\mathrm {\ GeV}}$	–
$\Delta \phi (\mathrm {jet_1},\vec {{p}}_\mathrm {T}^\mathrm {~miss})$	–	–	$>2.0$	–	–
$\Delta \phi (\tau ,\vec {{p}}_\mathrm {T}^\mathrm {~miss})$	–	–	$>1.0$	$>1.0$	$>1.0$
$E_{\mathrm {T}}^{\mathrm {miss}}/m_{\mathrm {eff}}$	–	–	$>0.2^{{*}} / 0.3^{{\dag }}$	$>0.2^{{*}} / 0.3^{{\dag }}$	$>0.2^{{*}} / 0.3^{{\dag }}$

**Fig. 4:**
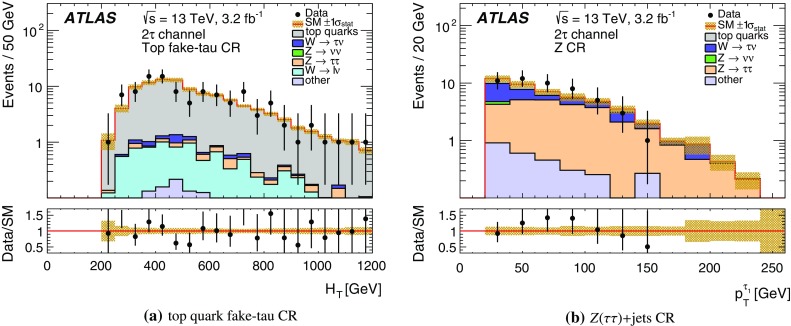
$H_{\mathrm {T}}$ distribution in the top quark fake-tau CR and transverse momentum of the leading tau lepton in the $Z(\tau \tau )$+jets CR of the $2\tau$ channel after the fit, illustrating the overall background modelling in the control regions. By construction, the total fitted background is equal to the number of observed events in each CR. The last bin includes overflow events. The *shaded bands* indicate the statistical uncertainties in the background predictions

In the $2\tau$ channel, the dominant backgrounds are $Z(\tau \tau )$+jets and dileptonic top quark contributions (including $t\bar{t}$ and single-top quark processes) with two true tau leptons, $W(\tau \nu )$+jets and semileptonic top quark contributions with one true and one fake tau lepton, and $W(\ell \nu )$+jets and top quark contributions with two fake tau leptons. Control regions are defined for *W*+jets and top quark backgrounds to extract normalisation factors related to the modelling of the process kinematics, and the modelling of real and fake tau leptons in the simulation. These CRs are separated from the SRs by replacing the requirement on the two tau leptons with requirements on different final-state objects, which stand in for true or fake tau leptons. To be independent from tau-lepton considerations, kinematic CRs are based on events with one muon, jets, $E_{\mathrm {T}}^{\mathrm {miss}}$, and without or with *b*-jets, to select $W(\mu \nu )$+jets and semileptonic top quark events with a final-state muon, respectively. The fake tau-lepton CRs use the same baseline selections as the kinematic CRs, but in addition, the presence of a loose tau-lepton candidate is required. Events with large $m_\mathrm {T}^\mu$ values are discarded to suppress the dileptonic top quark background with a muon and a true tau lepton. The true tau-lepton CRs, which target $W(\tau \nu )$+jets and semileptonic top quark processes with a true tau lepton, are based on events with a loose tau lepton, jets and $E_{\mathrm {T}}^{\mathrm {miss}}$, without or with *b*-jets. Similarly, contributions from fake tau leptons are suppressed by a requirement on $m_\mathrm {T}^\tau$. A separate CR is designed to study $Z(\tau \tau )$+jets events by inverting the $m_{\mathrm T}^{\tau _1}+m_{\mathrm T}^{\tau _2}$ and $H_{\mathrm {T}}$ requirements from the SRs. This selection requires two loose tau leptons of opposite electric charge. The selection criteria defining the various CRs are summarised in Table 5. Figure 4 illustrates the good background modelling in the CRs after the fit.

The VRs of the $2\tau$ channel are presented in Table 6. For the $Z(\tau \tau )$+jets background, the validation of the background extrapolation is performed from low-$H_{\mathrm {T}}$ to high-$H_{\mathrm {T}}$, while keeping the upper bound on $m_{\mathrm T}^{\tau _1}+m_{\mathrm T}^{\tau _2}$ which is effective in selecting $Z(\tau \tau )$+jets events. The validity of the top quark and $W(\tau \nu )$+jets background predictions obtained with alternative object selections are checked for selections with two reconstructed tau leptons. High values of $m_{\mathrm T}^{\tau _1}+m_{\mathrm T}^{\tau _2}$ are required to suppress $Z(\tau \tau )$+jets events as in the SRs, while upper bounds on $m_\text {T2}$ and $H_{\mathrm {T}}$ ensure there is no overlap between SRs and VRs. The same set of CRs and VRs is used for the three SRs of the $2\tau$ channel.

**Fig. 5:**
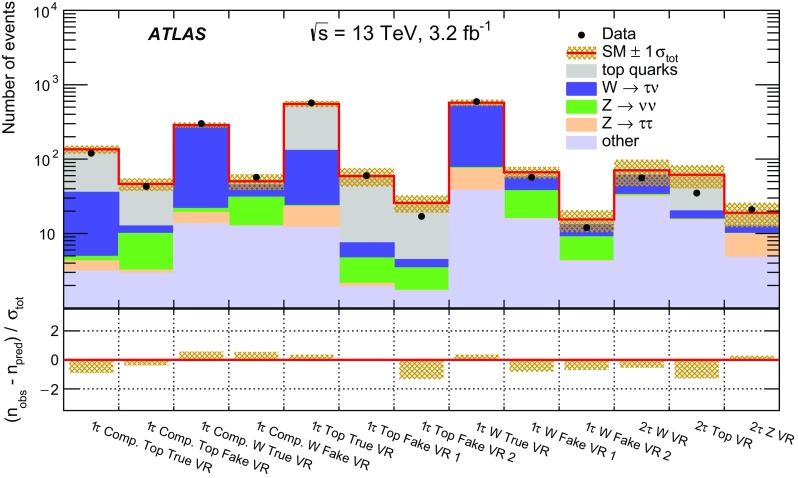
Number of observed events, $n_\mathrm {obs}$, and predicted background yields after the fit, $n_\mathrm {pred}$, in the validation regions of the $1\tau$ and $2\tau$ channels. The background predictions are scaled using normalisation factors derived in the control regions. The *shaded bands* indicate the statistical uncertainties in the background predictions, and correspond to the $\sigma _\mathrm {tot}$ uncertainties used in the *lower part* of the figure

The resulting normalisation factors for both channels do not deviate from 1 by more than 25%, except for $t\bar{t}$ events with fake tau leptons in the $1\tau$ channel, where the normalisation factor reaches 2 within large statistical uncertainty. The typical level of agreement between data and background distributions in the CRs after the fit can be seen in Figs. 3 and 4. Good modelling of kinematic distributions is observed in all CRs. The comparison between the number of observed events and the predicted background yields in the VRs is displayed in Fig. 5. Agreement within approximately one standard deviation is observed.

**Table 5:**
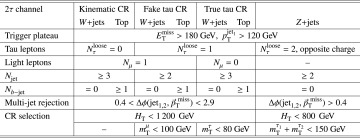
Overview of the control regions used in the $2\tau$ channel

**Table 6:**
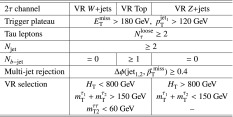
Overview of the validation regions defined in the $2\tau$ channel

### Multi-jet background

The multi-jet background contributes to the selection when two conditions are simultaneously fulfilled: jets have to be mis-identified as tau leptons, and large missing transverse momentum must arise from jet energy mis-measurement. This background is estimated from data, because final-state objects arising from mis-measurements are much more challenging to simulate than the reconstruction and identification of genuine objects. Moreover, the very large multi-jet production cross section at the LHC would imply simulating a prohibitively large number of multi-jet events.

The *jet smearing* method [81] employed in the $1\tau$ channel proceeds in two steps. First, multi-jet events with well-measured jets are selected in a data sample collected by single-jet triggers. This is achieved by requiring $E_{\mathrm {T}}^{\mathrm {miss}}/(\sum E_\mathrm {T})^{1/3} < 5 {\mathrm {\ GeV}}^{2/3}$, where the objects entering the $\sum E_\mathrm {T}$ term are those entering the $E_{\mathrm {T}}^{\mathrm {miss}}$ calculation. Selected events are required to have at least two jets, no light lepton and exactly one tau candidate satisfying the medium identification criteria. The selection is dominated by multi-jet production, such that most tau candidates are jets mis-identified as tau leptons. In a second step, jet energies are smeared according to the $p_{\text {T}}$-dependent jet energy resolution extracted from simulation. The smearing is performed multiple times for each event, leading to a large pseudo-data set where $E_{\mathrm {T}}^{\mathrm {miss}}$ originates from resolution effects and which includes an adequate fraction of jets mis-identified as tau leptons.

This method cannot be used in the $2\tau$ channel because of the limited number of events with well-measured jets that contain at least two loose tau candidates. Instead, a *fake rate* approach is adopted. The probability for jets to be mis-identified as tau leptons or muons, obtained from simulated dijet events, is applied to jets from an inclusive data sample collected by single-jet triggers and dominated by multi-jet events.

## Systematic uncertainties

For all simulated processes, theoretical and experimental systematic uncertainties are considered. The former includes cross-section uncertainties, which are not relevant for the dominant backgrounds normalised to data, and generator modelling uncertainties. The latter refers to all the uncertainties related to the reconstruction, identification, calibration and corrections applied to jets, tau leptons, electrons, muons and missing transverse momentum. Specific uncertainties are evaluated for the multi-jet background, which is estimated from data.

Theoretical uncertainties are evaluated for all simulated samples. For backgrounds that are normalised in CRs, the uncertainty in the transfer factors, i.e. the ratio of the expected event yields in a SR or VR over the respective CR, is evaluated for all SRs and VRs. The difference between the nominal simulation and the systematically varied sample is used as an additional uncertainty. For backgrounds that are evaluated from simulation alone, i.e. the diboson background, a global normalisation uncertainty is added. Uncertainties for *V*+jets samples generated with Sherpa are estimated by up and down variations by factors of two and one-half in the renormalisation and factorisation scales, resummation scale (maximum scale of the additional emission to be resummed by the parton shower) and CKKW matching scale (matching between matrix elements and parton shower). The effect of scale variations is parameterised at generator level as a function of the vector boson $p_{\text {T}}$ and the number of particle jets. For the top quark background, the nominal predictions from Powheg-Box + Pythia6 are compared with predictions from alternative generators, and the differences are taken as systematic uncertainties. The MG5_aMC@NLO + Herwig++ generators are used to evaluate uncertainties in the modelling of the hard scattering, parton shower and hadronisation. The Powheg-Box + Herwig++ generators are used to compute a specific uncertainty in the parton shower and hadronisation models. An uncertainty in the ISR modelling is also assessed by varying the Powheg-Box parameter which controls the transverse momentum of the first additional parton emission beyond the Born configuration. In the case of the diboson background, a 6% uncertainty in the cross section due to scale and PDF uncertainties is considered. Uncertainties in signal cross sections are obtained by using different PDF sets and factorisation and renormalisation scales, as discussed in Section 3.

Systematic uncertainties affecting jets arise from the jet energy scale and jet energy resolution [66], as well as efficiency corrections for jet-vertex-tagging [68] and *b*-tagging [82]. A set of $p_{\text {T}}$- and $\eta$-dependent uncertainties in the jet energy scale and resolution is estimated by varying the conditions used in the simulation. Another set of uncertainties accounts for the modelling of the residual pile-up dependence. Additional uncertainties account for the jet flavour composition of samples that are used to derive in-situ energy scale corrections, where jets are calibrated against well-measured objects. A punch-through uncertainty for jets not entirely contained in the calorimeters, as well as a single-hadron response uncertainty, are also included for high-$p_{\text {T}}$ jets. An overall uncertainty in the jet energy resolution is applied to jets in the simulation as a Gaussian energy smearing.

Systematic uncertainties affecting correctly identified tau leptons arise from the reconstruction, identification and tau-electron overlap-removal efficiencies, and the energy scale calibration [75]. Most of the uncertainties are estimated by varying nominal parameters in the simulation: detector material, underlying event, hadronic shower model, pile-up and noise in the calorimeters. The uncertainty in the energy scale also includes non-closure of the calibration found in simulation, a single-pion response uncertainty, and an uncertainty in the in-situ energy calibration of data with respect to simulation derived in $Z(\tau \tau )$ events with a hadronically decaying tau lepton and a muon in the final state. In the case of signal samples, which undergo fast calorimeter simulation, a dedicated uncertainty takes into account the difference in performance between full and fast simulation. The effect of mis-identified tau leptons is largely constrained by the background estimation approaches. Uncertainties arise due to the extrapolation from the CRs to the VRs and SRs. These are considered as part of the theory uncertainties, which account for the impact of hadronisation on the mis-identification of jets as tau leptons.

Systematic uncertainties affecting electrons and muons are related to the energy or momentum calibration, as well as efficiency corrections for the reconstruction, identification and isolation requirements. These uncertainties have a negligible impact on the background predictions.

Systematic uncertainties in the missing transverse momentum originate from uncertainties in the energy or momentum calibration of jets, tau leptons, electrons, and muons, which are propagated to the $E_{\mathrm {T}}^{\mathrm {miss}}$ calculation. Additional uncertainties are related to the calculation of the track-based soft-term. These uncertainties are derived by studying the $p_{\text {T}}$ balance in $Z(\mu \mu )$ events between the soft-term and the hard-term composed of all reconstructed objects. Soft-term uncertainties include scale uncertainties along the hard-term axis, and resolution uncertainties along and perpendicular to the hard-term axis [83].

A systematic uncertainty of the pile-up modelling is estimated by varying the distribution of the average number of interactions per bunch crossing in the simulation. The range of the variation is determined by studying the correlation in data and simulation between the average number of interactions and the number of reconstructed primary vertices. This uncertainty ranges from a few percent in the $1\tau$ channel to about 15% in the poorly populated SRs of the $2\tau$ channel.

The uncertainty in the integrated luminosity is $\pm 2.1$%. It is derived, following a methodology similar to that detailed in Ref. [84], from a calibration of the luminosity scale using *x*–*y* beam-separation scans performed in August 2015. This uncertainty only affects the diboson background prediction and the signal yields, as other backgrounds are normalised to the data.

The systematic uncertainty in the small multi-jet background contribution is estimated by considering alternative normalisation regions and different jet smearing parameters in the case of the $1\tau$ channel. An uncertainty of the order of 70% is found for the $1\tau$ channel, and a 100% uncertainty is assigned in the $2\tau$ channel.

The influence of the main systematic uncertainties in the total background predictions in the SRs of the $1\tau$ and $2\tau$   channels are summarised in Table 7. The uncertainties reported in the table are derived assuming that no signal is present in the CRs.

The total uncertainties range between 13 and 72%. For all SRs, the largest uncertainties, between 7 and 60%, originate from the MC generator modelling of top quark events. The larger uncertainty found for the Compressed SR of the $2\tau$ channel is explained by the larger statistical uncertainty in the predictions from alternative generators. Energy scale uncertainties affecting tau leptons and jets contribute significantly in all regions as well. Other uncertainties, e.g. in the *b*-tagging efficiency and the jet energy resolution, do not play a large role in most of the SRs.

**Table 7:** Dominant systematic uncertainties in the total background predictions, in percent, for the signal regions of the $1\tau$ (top) and $2\tau$ (bottom) channels. The total systematic uncertainty accounts for other minor contributions not listed in this table, as well as correlations between the uncertainties

Source of uncertainty	$1\tau$ Compressed SR	$1\tau$ Medium-Mass SR	$1\tau$ High-Mass SR
Top generator modelling	8.1	6.5	12
*V*+jets generator modelling	1.5	6.4	6.3
Jet energy scale	2.0	6.7	0.4
Jet energy resolution	0.6	0.2	0.7
*b*-tagging efficiencies	1.9	3.2	7.7
Tau energy scale	1.8	2.8	5.5
Total	13	16	21
Source of uncertainty	$2\tau$ Compressed SR	$2\tau$ High-Mass SR	$2\tau$ GMSB SR
Top generator modelling	60	23	22
*V*+jets generator modelling	4.2	6.3	4.3
Jet energy scale	14	2.0	6.0
Jet energy resolution	8.1	1.2	4.3
*b*-tagging efficiencies	8.8	5.1	7.7
Tau energy scale	19	13	8.5
Total	72	36	35

## Results

**Fig. 6:**
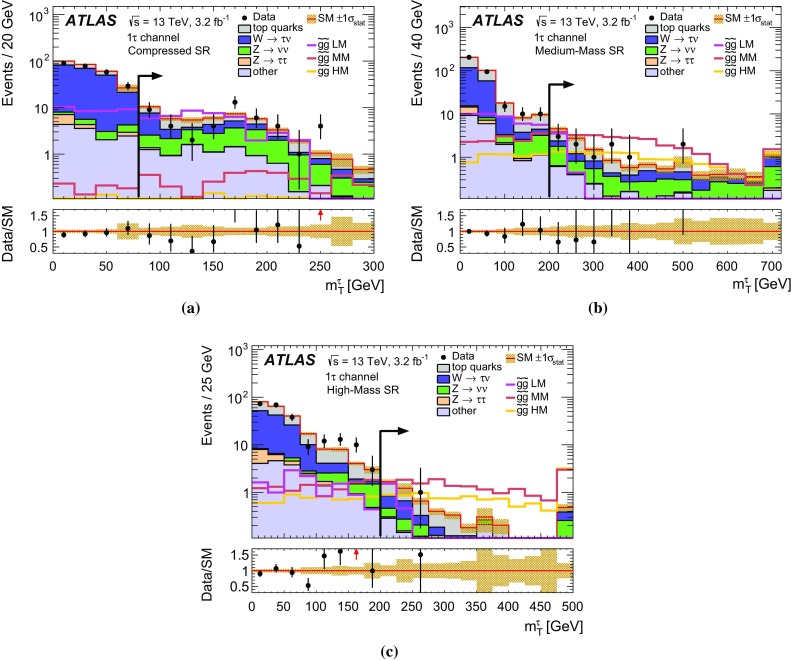
$m_\mathrm {T}^\tau$ distributions for “extended SR selections” of the $1\tau$ channel, for **a** the Compressed SR selection without the $m_{\mathrm {T}} ^\tau > 80{\mathrm {\ GeV}}$ requirement, **b** the Medium-Mass SR selection without the $m_{\mathrm {T}} ^\tau > 200{\mathrm {\ GeV}}$ requirement, and **c** the High-Mass SR selection without the $m_{\mathrm {T}} ^\tau >200 {\mathrm {\ GeV}}$ requirement. The last bin includes overflow events. The *shaded bands* indicate the statistical uncertainties in the background predictions. *Red arrows* in the data/SM ratio indicate bins where the corresponding entry falls outside the plotted range. The signal region is indicated by the *black arrow*. Signal predictions are overlaid for several benchmark models, normalised to their predicted cross sections. For the simplified model, *LM* low mass splitting, or compressed scenario, with $m_{\tilde{g}}=665 {\mathrm {\ GeV}}$ and $m_{\tilde{\chi }_1^0}=585 {\mathrm {\ GeV}}$; *MM* medium mass splitting, with $m_{\tilde{g}}=1145 {\mathrm {\ GeV}}$ and $m_{\tilde{\chi }_1^0}=265 {\mathrm {\ GeV}}$; *HM* high mass splitting scenario, with $m_{\tilde{g}}=1305 {\mathrm {\ GeV}}$ and $m_{\tilde{\chi }_1^0}=105 {\mathrm {\ GeV}}$

**Fig. 7:**
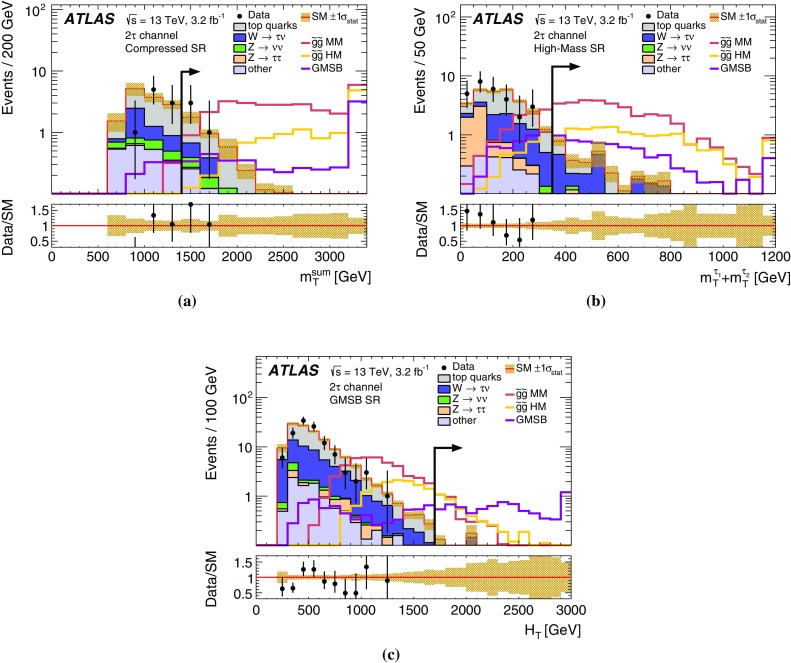
Kinematic distributions for “extended SR selections” of the $2\tau$ channel, for **a** $m_{\mathrm {T}} ^\mathrm {sum}$ in the Compressed SR selection without the $m_{\mathrm {T}} ^\mathrm {sum}>1400 {\mathrm {\ GeV}}$ requirement, **b** $m_{\mathrm T}^{\tau _1}+m_{\mathrm T}^{\tau _2}$ in the High-Mass SR selection without the $m_{\mathrm T}^{\tau _1}+m_{\mathrm T}^{\tau _2} >350{\mathrm {\ GeV}}$ requirement, and **c** $H_{\mathrm {T}}$ in the GMSB SR selection without the $H_{\mathrm {T}} > 1700 {\mathrm {\ GeV}}$ requirement. The last bin includes overflow events. The *shaded bands* indicate the statistical uncertainties in the background predictions. The signal region is indicated by the *black arrow*. Signal predictions are overlaid for several benchmark models, normalised to their predicted cross sections. For the simplified model, *MM* medium mass splitting, with $m_{\tilde{g}}=1145 {\mathrm {\ GeV}}$ and $m_{\tilde{\chi }_1^0}=265 {\mathrm {\ GeV}}$; *HM* high mass splitting scenario, with $m_{\tilde{g}}=1305 {\mathrm {\ GeV}}$ and $m_{\tilde{\chi }_1^0}=105 {\mathrm {\ GeV}}$. The GMSB benchmark model corresponds to $\Lambda = 90{\mathrm {\ TeV}}$ and $\tan \beta =40$

Kinematic distributions for *extended SR selections* of the $1\tau$ and $2\tau$ channels are shown in Figs. 6 and  7, respectively. These regions are defined by the same set of selection criteria as for the SRs, except that the criterion corresponding to the plotted variable is not applied. Data and background predictions, fitted to data in the control regions, are compared, and signal predictions are also shown for several benchmark models. Variables providing the most discrimination between signal and background are displayed: $m_{\mathrm {T}} ^\tau$ distributions for the $1\tau$ channel, and $m_{\mathrm {T}} ^\mathrm {sum}$, $m_{\mathrm T}^{\tau _1}+m_{\mathrm T}^{\tau _2}$ and $H_{\mathrm {T}}$ distributions for the $2\tau$ channel. Reasonable agreement between data and background distributions is observed, given the low event yields remaining in data after these selections. The numbers of observed events and expected background events in all SRs are reported in Tables 8 and 9 for the $1\tau$ and $2\tau$ channels, respectively.

No significant excess is observed in data over the SM predictions. Therefore, upper limits are set at the 95% confidence level (CL) on the number of hypothetical signal events, or equivalently, on the signal cross section. The one-sided profile-likelihood-ratio test statistic is used to assess the compatibility of the observed data with the background-only and signal-plus-background hypotheses. Systematic uncertainties are included in the likelihood function as nuisance parameters with Gaussian probability densities. Following the standards used for LHC analyses, *p*-values are computed according to the $\mathrm {CL_s}$ prescription [85].

Model-independent upper limits are calculated for each SR, assuming no signal contribution in the CRs. The results are derived using profile-likelihood-ratio distributions obtained from pseudo-experiments. Upper limits on signal yields are converted into limits on the visible cross section ($\sigma _\mathrm {vis}$) of BSM processes by dividing by the integrated luminosity of the data. The visible cross section is defined as the product of production cross section, acceptance and selection efficiency. Results are summarised at the bottom of Tables 8 and 9. The most stringent observed upper limits on the visible cross section are $1.17~\mathrm {fb}$ for the High-Mass SR of the $1\tau$ channel and $1.07~\mathrm {fb}$ for the High-Mass and GMSB SRs of the $2\tau$ channel.

**Table 8:** Number of observed events and predicted background yields in the three signal regions of the $1\tau$  channel. The background prediction is scaled using normalisation factors derived in the control regions. All systematic and statistical uncertainties are included in the quoted uncertainties. The bottom part of the table shows the observed and expected model-independent upper limits at the 95% CL on the number of signal events, $S_\mathrm{obs}^{95}$ and $S_\mathrm{exp}^{95}$, respectively, the corresponding observed upper limit on the visible cross section, $\langle \mathrm{\sigma _{vis}}\rangle _\mathrm{obs}^{95}$, and the $\mathrm {CL_b}$ value, i.e. the confidence level observed for the background-only hypothesis

$1\tau$ channel	Compressed SR	Medium-Mass SR	High-Mass SR
Data	47	11	1
Total background	$49.2 \pm 6.2$	$15.0 \pm 2.4$	$5.7 \pm 1.2$
Top	$14.3 \pm 4.5$	$6.0 \pm 1.3$	$2.49 \pm 0.87$
$W(\tau \nu )$+jets	$12.1 \pm 1.3$	$2.78 \pm 0.62$	$1.17 \pm 0.33$
$Z(\nu \nu )$+jets	$13.9 \pm 2.3$	$3.8 \pm 1.1$	$0.83 \pm 0.21$
*V*+jets, other	$6.24 \pm 0.90$	$1.44 \pm 0.32$	$0.75 \pm 0.23$
Diboson	$1.85 \pm 0.23$	$0.76 \pm 0.16$	$0.20 \pm 0.03$
Multi-jet	$0.74 \pm 0.54$	$0.19 \pm 0.18$	$0.24 \pm 0.17$
$S_\mathrm{obs}^{95}$ ($S_\mathrm{exp}^{95}$)	16.7 (${18.4}^{+6.9}_{-5.0}$)	7.5 (${9.7}^{+3.5}_{-2.5}$)	3.8 (${6.1}^{+2.1}_{-1.5}$)
$\langle \mathrm{\sigma _{vis}}\rangle _\mathrm{obs}^{95}$ (fb)	5.19	2.34	1.17
$\mathrm {CL_b}$	0.41	0.23	0.02

**Table 9:** Number of observed events and predicted background yields in the three signal regions of the $2\tau$ channel. The background prediction is scaled using normalisation factors derived in the control regions. All systematic and statistical uncertainties are included in the quoted uncertainties. The bottom part of the table shows the observed and expected model-independent upper limits at the 95% CL on the number of signal events, $S_\mathrm{obs}^{95}$ and $S_\mathrm{exp}^{95}$, respectively, the corresponding observed upper limit on the visible cross section, $\langle \mathrm{\sigma _{vis}}\rangle _\mathrm{obs}^{95}$, and the $\mathrm {CL_b}$ value, i.e. the confidence level observed for the background-only hypothesis

$2\tau$ channel	Compressed SR	High-Mass SR	GMSB SR
Data	4	0	0
Total background	$4.2 \pm 3.0$	$3.2 \pm 1.2$	$0.69 \pm 0.24$
Top	$2.5_{-2.5}^{+2.9}$	$0.87 \pm 0.78$	$0.20 \pm 0.20$
$W(\tau \nu )$+jets	$0.51 \pm 0.38$	$1.75 \pm 0.65$	$0.31 \pm 0.14$
$Z(\tau \tau )$+jets	$0.04 \pm 0.02$	$0.13 \pm 0.06$	$0.04 \pm 0.02$
$Z(\nu \nu )$+jets	$0.28 \pm 0.12$	$0.07 \pm 0.03$	$0.02 \pm 0.01$
$W(\ell \nu )$+jets	$0.37 \pm 0.34$	$0.12 \pm 0.07$	$0.02 \pm 0.01$
Diboson	$0.25 \pm 0.10$	$0.21 \pm 0.08$	$0.06 \pm 0.02$
Multi-jet	$0.21 \pm 0.21$	$0.07 \pm 0.07$	$0.06 \pm 0.06$
$S_\mathrm{obs}^{95}$ ($S_\mathrm{exp}^{95}$)	8.2 (${8.0}^{+2.1}_{-2.0}$)	3.4 (${4.8}^{+1.4}_{-1.0}$)	3.4 (${3.7}^{+0.5}_{-0.2}$)
$\langle \mathrm{\sigma _{vis}}\rangle _\mathrm{obs}^{95}$ (fb)	2.55	1.07	1.07
$\mathrm {CL_b}$	0.53	0.12	0.53

**Fig. 8:**
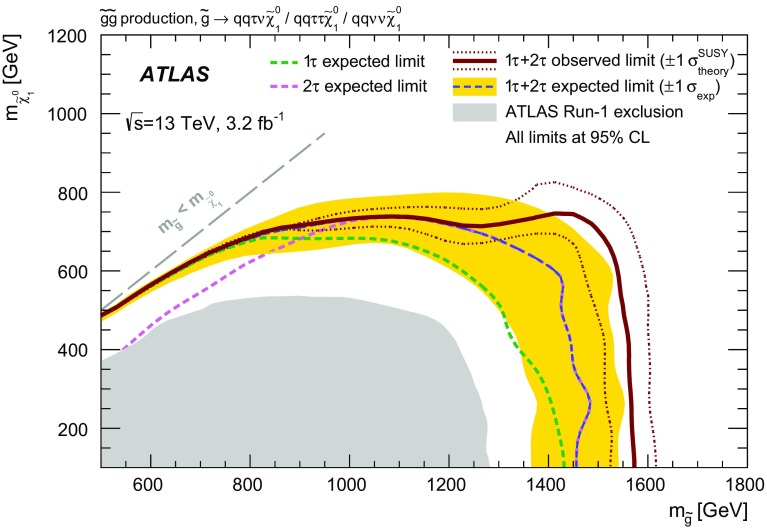
Exclusion contours at the 95% confidence level for the simplified model of gluino pair production, based on results from the $1\tau$ and $2\tau$ channels. The *red solid line* and the *blue dashed line* correspond to the observed and median expected limits, respectively, for the combination of the two channels. The *yellow band* shows the one-standard-deviation spread of expected limits around the median. The effect of the signal cross-section uncertainty in the observed limits is shown as *red dotted lines*. Additionally, expected limits are shown for the $1\tau$ and $2\tau$ channels individually as *dashed green* and *magenta lines*, respectively. The previous ATLAS result [19] obtained with 20.3 ${\mathrm{{fb}^{-1}}}$ of $8 {\mathrm {\ TeV}}$ data is shown as the *grey filled area*

**Fig. 9:**
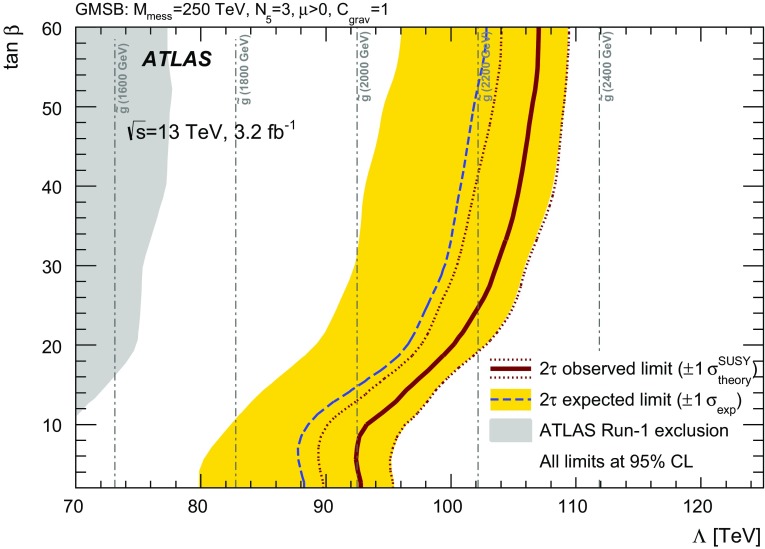
Exclusion contours at the 95% confidence level for the gauge-mediated supersymmetry-breaking model, based on results from the $2\tau$ channel. The *red solid line* and the *blue dashed line* correspond to the observed and median expected limits, respectively. The *yellow band* shows the one-standard-deviation spread of expected limits around the median. The effect of the signal cross-section uncertainty in the observed limits is shown as *red dotted lines*. The previous ATLAS result [14] obtained with 20.3 ${\mathrm{{fb}^{-1}}}$ of $8 {\mathrm {\ TeV}}$ data is shown as the *grey filled area*

The results are interpreted in the context of the simplified model of gluino pair production and the GMSB model. In the case of model-dependent interpretations, the signal contribution in the control regions is included in the calculation of upper limits, and asymptotic properties of test-statistic distributions are used [86]. Exclusion contours at the 95% CL are derived in the $(m_{\tilde{g}},m_{\tilde{\chi }_1^0})$ plane for the simplified model and in the $(\Lambda ,\tan \beta )$ plane for the GMSB model. Results are shown in Figs. 8 and 9. The solid lines and the dashed lines correspond to the observed and median expected limits, respectively. The band shows the one-standard-deviation spread of the expected limits around the median, which originates from statistical and systematic uncertainties in the background and signal. The theoretical uncertainty in the signal cross section is not included in the band. Its effect on the observed limits is shown separately as the dotted lines. For the simplified model, exclusion contours are shown for the $1\tau$ and $2\tau$ channels and their combination. The combination is performed by selecting, for each signal scenario, the SR with the lowest expected $\mathrm {CL_s}$ value. The combination retains the Compressed SR of the $1\tau$ channel in the region where the LSP mass is close to the gluino mass, and favours the High-Mass SR of the $2\tau$ channel when the mass splitting is large. For the GMSB model, limits are shown for the GMSB SR of the $2\tau$ channel. The stronger limits at high values of $\tan \beta$ are explained by the nature of the NSLP, which is the lightest scalar tau in this region. For both models, the exclusion limits obtained with 3.2 ${\mathrm{{fb}^{-1}}}$ of collision data at $\sqrt{s}=13 {\mathrm {\ TeV}}$ significantly improve upon the previous ATLAS results [14, 19] established with 20.3 ${\mathrm{{fb}^{-1}}}$ of 8 ${\mathrm {\ TeV}}$ data.

## Summary

A search for squarks and gluinos has been performed in events with hadronically decaying tau leptons, jets and missing transverse momentum, using 3.2 ${\mathrm{{fb}^{-1}}}$ of *pp* collision data at $\sqrt{s}=13$ TeV recorded by the ATLAS detector at the LHC in 2015. Two channels, with either one tau lepton or at least two tau leptons, are separately optimised. The numbers of observed events in the different signal regions are in agreement with the Standard Model predictions. Results are interpreted in the context of a gauge-mediated supersymmetry breaking model and a simplified model of gluino pair production with tau-rich cascade decay. In the GMSB model, limits are set on the SUSY-breaking scale $\Lambda$ as a function of $\tan \beta$. Values of $\Lambda$ below $92 {\mathrm {\ TeV}}$ are excluded at the 95% CL, corresponding to gluino masses below $2000 {\mathrm {\ GeV}}$. A stronger exclusion is achieved for large values of $\tan \beta$, where $\Lambda$ and gluino mass values are excluded up to $107 {\mathrm {\ TeV}}$ and $2300 {\mathrm {\ GeV}}$, respectively. In the simplified model, gluino masses are excluded up to $1570 {\mathrm {\ GeV}}$ for neutralino masses around $100 {\mathrm {\ GeV}}$, neutralino masses below $700 {\mathrm {\ GeV}}$ are excluded for all gluino masses between 800 and $1500 {\mathrm {\ GeV}}$, while the strongest neutralino-mass exclusion of $750 {\mathrm {\ GeV}}$ is achieved for gluino masses around $1450 {\mathrm {\ GeV}}$. A dedicated signal region provides good sensitivity to scenarios with a small mass difference between the gluino and the neutralino LSP.

## Footnotes

1 ATLAS uses a right-handed coordinate system with its origin at the nominal interaction point in the centre of the detector and the *z*-axis along the beam pipe. The *x*-axis points from the interaction point to the centre of the LHC ring and the *y*-axis points upward. Cylindrical coordinates $(r,\phi )$ are used in the transverse plane, $\phi$ being the azimuthal angle around the beam pipe. The pseudorapidity is defined in terms of the polar angle $\theta$ as $\eta =-\ln \tan (\theta /2)$.
